# Electric ambulances: will the need for charging affect response times?

**DOI:** 10.1007/s10729-026-09786-2

**Published:** 2026-09-01

**Authors:** Nanne A. Dieleman, Caroline J. Jagtenberg

**Affiliations:** Department of Operations Analytics, Vrije Universiteit Amsterdam, De Boelelaan 1095-1105, 1081HV Amsterdam, The Netherlands

**Keywords:** Emergency medical services, Battery electric vehicles, Discrete-event simulation, Ambulances, Urgent patient transport

## Abstract

Ambulance providers currently rely on diesel vehicles for patient transport in their daily operations, but the first providers will likely transition to electric vehicles in the coming years. Prior to this transition, they would want to assess whether the non-negligible charging times impact response times. Our paper introduces this novel application and performs a study focused on range and charging infrastructure. We developed ELASPY, an open-source decision support system based on discrete-event simulation built in Python, to simulate the emergency response process, and used it to predict under which battery capacities and charger locations an electric ambulance fleet would achieve response times comparable to the equivalent diesel fleet. We also include a simulation-based optimization framework for charger allocation. We present a case study for the province of Utrecht, the Netherlands, from which derive managerial insights for ambulance providers. Our results did not reveal concerning signals for daily operations under the expected operating conditions. However, the sting is in the tail, and even ambulance providers with a low busyness will have to think about calamities such as prolonged power outages. We encourage decision makers to insert their own historical incident data into ELASPY and assess how transitioning from diesel to electric ambulance vehicles would affect response times in their region. The software is publicly available including documentation and set-up guidelines.

## Introduction

Although the transition to electric vehicles has gained momentum across various sectors, its implementation within Emergency Medical Services (EMS), and notably the integration of electric ambulances, is only in its nascent stages. To date, to the best of our knowledge, no ambulance provider uses electric vehicles for patient transport in their daily operations. However, in several countries, practitioners are planning to purchase such vehicles, including the UK [25] and USA [16]. With merely pilot tests underway [46, 66], the world is starting to notice that a widespread adoption of electric ambulances faces unique challenges, including the infrastructure required to support their operation.

Despite the growing interest in the use of Battery Electric Vehicles (BEVs) within EMS, the topic has not yet been addressed in the literature. This presents a timely and pertinent avenue for exploration. Our paper bridges the gap by synthesizing existing research on traditional ambulance planning and exploring analogous applications of BEVs in other industries.

Given that BEV charging times are non-negligible, an important concern is whether the transition can be made without negatively affecting patient care. In ambulance planning, it is common to translate the desire to have a high level of patient care into response-time KPIs. Although creators of such KPIs are generally aware that some aspects are lost in this translation, the field is laden with response-time targets. Hence, understandably, EMS providers are reluctant to transition to an electric fleet as long as they are unsure how the charging process would affect their response times. After all, ‘putting its goals for net zero above patient safety’ [56] is a news item that any EMS operator would undoubtedly prefer to avoid.

The ability of electric ambulances to stay sufficiently charged depends significantly on the availability and location of charging stations. Particular to the EMS industry is that they are unlikely to accept a plan that includes the use of public chargers. One reason is that some countries’ regulations mandate that the crew must rest at a base when not attending to a patient. Another reason is that ambulance providers nowadays often struggle with staffing shortages, making managers hesitant to introduce additional tasks that would make working on the ambulance less attractive. Combine this with the fact that ambulances often stand still at hospitals while the crew is dropping off a patient, it raises the question whether one can build chargers - solely for ambulance use and never occupied by regular cars - at hospitals. Keeping in mind that the hospital and the EMS provider are often two different entities, this is a nontrivial decision. However, in anticipation of such changes, some ambulance providers are already proving proactive: The Dutch UMCG Ambulancezorg had hospitals in their region sign a letter of intent stating that they would help realize the necessary infrastructure for electric ambulances [60].

Against this backdrop emerges the central research question of our study: how to determine the number and location of charging stations such that an electric ambulance fleet can achieve response times comparable to the equivalent diesel fleet? Addressing this question necessitates a comprehensive understanding of the interplay between charging infrastructure, vehicle deployment strategies, and operational logistics within the EMS context. To accurately reflect the stochasticity and interdepencies in the system, we developed an open-source decision support system based on discrete-event simulation built in Python called Electric Ambulance Simulator Python (ELASPY) for electric ambulance operations. ELASPY can be used to evaluate specific scenarios, but also to perform simulation-based optimization.

To demonstrate the software, this paper includes a case study of the region Utrecht, the Netherlands. Our managerial insights derived from this case study were well received by practitioners. Insights that stretch beyond this region can be obtained by (1) our study of how the conclusions change when system parameters change, and (2) the fact that we make our code available to the public. Decision makers can therefore use ELASPY by providing their data as input to analyze whether it is feasible to transfer to electric ambulances and if so, under which conditions.

The authors are in contact with a network of Dutch EMS providers called Axira [7] that was founded in 2009 and covers, at the time of writing, more than one third of the country [8]. Axira aims to facilitate complex innovation that might be economically prohibitive for any single entity to undertake alone, the electrification of the ambulance fleet being an important example. Our contacts in Axira helped us identify meaningful questions. In addition, they estimated realistic parameter values regarding the battery capacity and energy consumption of electric ambulances, which we also provide in this paper. Recently, the knowledge acquired by Axira was elevated to a national level by transferring it to Ambulancezorg Nederland [5].

The contribution of this paper is threefold. First, it introduces an open-source software package for simulating electric ambulances, complemented by an optimization method for charger allocation. Second, it presents a case study for the region of Utrecht, the Netherlands, providing managerial insights for practitioners considering the transition to electric ambulances. Third, it introduces the Operations Research community to the emerging topic of ambulance fleet electrification and highlights avenues for further research on related planning challenges.

The remainder of this paper is structured as follows. In Section 2, we discuss related work. Section 3 describes the simulation model as well as the data used to model Utrecht. Section 4 contains the results of our case study and we end with a discussion and conclusion in Section 5.

## Related work

Using electric vehicles for EMS is a relatively new topic, and therefore the literature is extremely scarce. There is effectively only Rigas et al. [53], discussing a number of high-level research questions related to the efficient deployment of electric ambulances; without attempting to answer them, and Rottkamp et al. [55], a conference proceeding that investigates how so-called dynamic ambulance redeployment should be changed when vehicles are electric.

This section continues by (1) summarizing the literature on traditional ambulance planning, and (2) highlighting articles that model BEVs in other applications, including public transport, taxis, and last-mile deliveries.

### Traditional ambulance planning

Ambulance providers typically measure their performance in terms of response times: the duration between a call arriving in the call center and an ambulance arriving on-site. Targets are often phrased as service level agreements, for example, in the Netherlands 95% of the most urgent patients should be reached within 15 minutes [21].

The decisions involved in ambulance operations can be divided into three different planning stages: strategic, tactical, and operational. The *strategic* level deals with long-term decisions such as the opening and closing of base locations, the purchase of vehicles, and the hiring and firing of staff. Most operations research literature in this category focuses on the base locations and their relation to response times. The *tactical* level deals with the medium-long term, which includes decisions such as how many vehicles to position at each base. The number of vehicles per base is intertwined with the strategic decision of where the bases are, therefore most modern literature takes both these decisions simultaneously. A review of EMS facility location models covering both the strategic and tactical level can be found in Li et al. [41]. The *operational* planning involves day-to-day or even real-time decisions. The latter includes dispatch decisions [31], which hospital to choose and where to send idle vehicles, also known as dynamic ambulance redeployment [22, 24, 30, 45, 75].

We observe that approaching problems through computer simulation is more common on the operational level than on the other planning levels. Henderson and Mason [27] discuss research models and methods for simulating and optimizing ambulance operations. Recently, an open-source ambulance optimization package written in Julia was published [52]. Another topic that has recently gained attention through simulation is ambulance services complemented by volunteer help [34, 64].

The remainder of this section deals with BEVs in other industries. Since that literature is extensive, we only highlight the most relevant articles, focusing in particular on the optimization of routing and charging decisions.

### Public Transportation

Electric vehicles are becoming increasingly common in public transportation, and especially Battery Electric Buses (BEBs) are popular. For example, the share of electric buses in the Netherlands has increased from 4.5% in 2019 to 18.4% in 2022 [15]. BEBs are often charged by fast chargers that require high power.

The plannable nature of buses leaves room for optimizing a charging schedule. This is, for example, done by Leou and Hung [39], Jahic et al. [32], Abdelwahed et al. [3], He et al. [26]. Abdelwahed et al. [3] minimize charging costs and the impact on the grid using Mixed Integer Linear Programming (MILP). He et al. [26] formulate a nonlinear non-convex charging problem and then reformulate and discretize it to obtain a linear model. Yang et al. [73] investigate wireless charging while on the road and optimize a charging schedule for this purpose.

The positioning of charging stations in the bus network is also studied [6, 42, 70–72]. Kunith et al. [38] and Liu and Song [43] jointly optimize the positioning of the charging stations and the required battery capacity of the buses. Xylia et al. [71] develop a dynamic optimization model for electric bus charging infrastructure by combining geospatial analysis with a MILP. This paper is further extended in Xylia et al. [72] which includes charging time constraints. Finally, some models combine the development of charging schedules and the positioning of charging stations [28, 54, 69].

Buses have at least two characteristics that make electrification easier than for ambulances. First, buses drive fixed and pre-determined routes. In contrast, ambulances serve unscheduled events, which makes it harder to plan the required capacity and charging schedule upfront. Second, BEBs are often charged by fast chargers, while fast chargers may not be available in the case of ambulances due to high purchasing costs or other restrictions such as fire safety at hospitals. Slow chargers create a more constrained problem, which makes optimization more important.

### Electric taxis

A considerable amount of research has been done in the area of electric taxis. Taxi trip data from New York City is frequently used in studies [29, 37, 57], perhaps because of its widespread accessibility [33, 48]. Many papers study the placement of taxi charging stations [12, 18, 40, 50, 59, 74]. Several of these papers use GPS data to study travel patterns.

An important problem in the taxi context is ride hailing, in which an operator assigns requests to vehicles while individual drivers decide on charging and repositioning. The drivers being selfish agents means the problem is often approached from a game-theoretical perspective, where the task becomes to find equilibria. Different solution methods are used, including reinforcement learning [37, 57], and simulation [77].

An electric taxi fleet controlled by a central operator is perhaps the problem that is most similar to electric ambulance planning. However, there are a few differences. First, even if taxi *dispatching* is centralized, the drivers still often decide to *reposition* on their own, which makes planning more difficult. Second, ambulance rides are urgent: if ambulances are not sufficiently charged, it can mean the difference between life and death. Moreover, many ambulance providers face targets that are prescribed by law. Failing to meet them can lead to severe fines or even losing their license to operate. Finally, a typical goal in the taxi industry is to maximize profit, with the option to ignore customers who are difficult to reach or present themselves at an inconvenient time. The ambulance industry offers no such option, meaning that a decision-maker has little to no influence on the system’s busyness.

### Last-mile logistics

In recent years, the field of last-mile logistics has seen an increase in electric vehicle usage and the corresponding need for route planning tools that incorporate charging. Dalmijn et al. [19] optimize a charging scheduling of EVs for the last-mile distribution of an e-grocer, taking into account battery degradation and time-dependent energy costs. Kin et al. [35] evaluates charging strategies for urban freight transport, and compares the costs of purchasing large batteries (sufficient to finish an entire route) against having a smaller battery plus opportunity charging costs. The mathematical models in this field are typically either electric vehicle routing or team orienteering problems, as reviewed in Martins et al. [44]. These last-mile logistics differ from EMS, because EMS requests occur random, so the concept of route planning is not relevant. Moreover, in last-mile logistics minimizing the distance traveled is important, whereas in EMS the focus is on minimizing response times.

### Concluding remarks

An extensive literature exists on the electrification of vehicles in different domains. However, the ambulance sector differs from these domains in several discussed ways; the most prominent being that lives are at stake. Therefore, it is essential to ensure that shifting to electric vehicles does not lead to worse response times. Moreover, ambulances need to be able to operate under the most demanding circumstances including disasters. This is more difficult if charging is involved, since this may lead to long periods during which ambulances cannot be used. Therefore, the electrification of ambulances calls for studies that explicitly consider the influence electric vehicles and in particular charging has on the ambulance emergency system. In the subsequent sections, we discuss the developed simulator and the performed case study.

## Methods and data

In this section, we first argue why we chose to answer our research question by simulation. We then give a description of the developed models and the data used for our case study.

### Justification

When it comes to charging decisions, we agree with Unterluggauer et al. [61] that such a study requires (1) detailed modeling of charging demand that accounts for randomness and variability in space and time rather than averages, (2) the need to consider a mix of different types of charging options rather than only fast charging and (3) the need to use large-scale and real-world case studies. We emphasize that, in this context, synthetic models and abstractions from the real world are unlikely to be of value for decision makers.

The current models in the EMS literature are not directly suitable for analyzing a fleet of electric ambulances. One reason is that they typically do not consider kilometers driven (with and without patient on board). This was not necessary because refueling decisions were not part of the models. When transitioning to an electric fleet, however, the distance driven has a direct impact on the state of charge, whether a next destination can be reached, and how long the vehicle should remain at the charging station.

While the crucial decision of where to build chargers is a strategic one, we have little hope of successfully using or extending existing strategic (and tactical) models. Those models struggle to accurately capture when and where ambulance vehicles will be available. They often include simplifying assumptions, such as independence between vehicles or ambulances always responding from a base. However, the busier the systems, the less accurate these assumptions are. Moreover, these assumptions become more limiting and less realistic for electric vehicles. The spatial and temporal relationships of the system simply have to be taken into account.

**Fig. 1:**
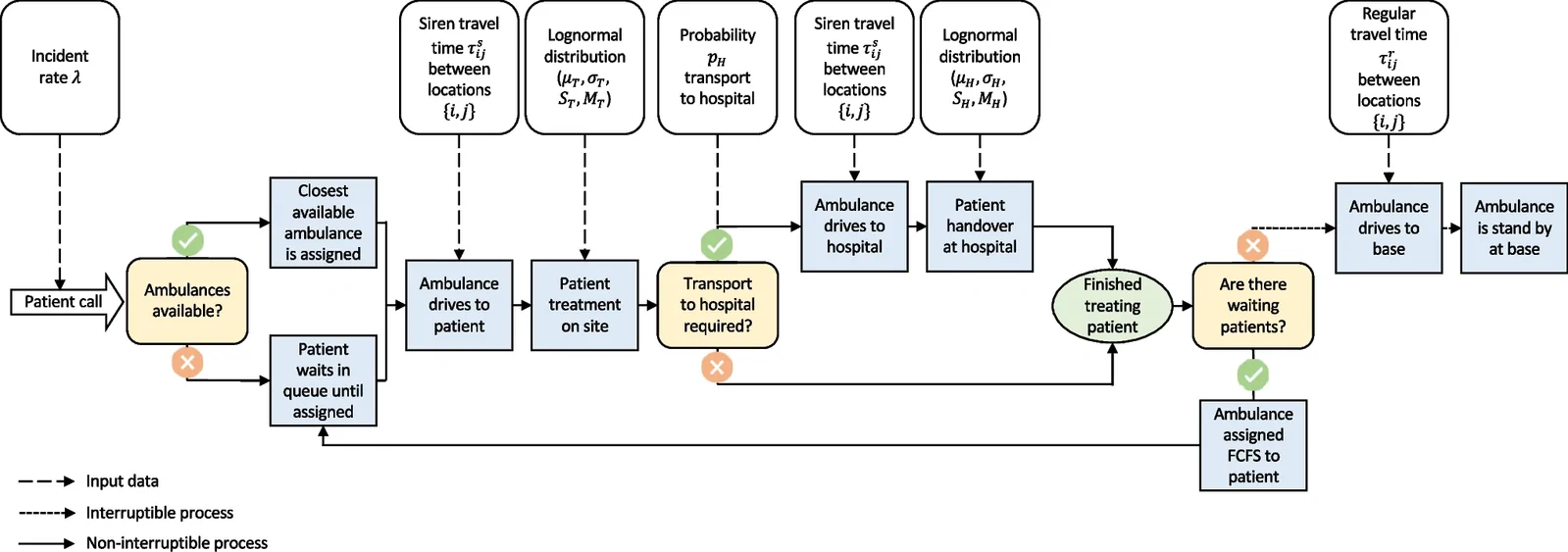
Overview of the process for the diesel ambulance simulation

**Fig. 2:**
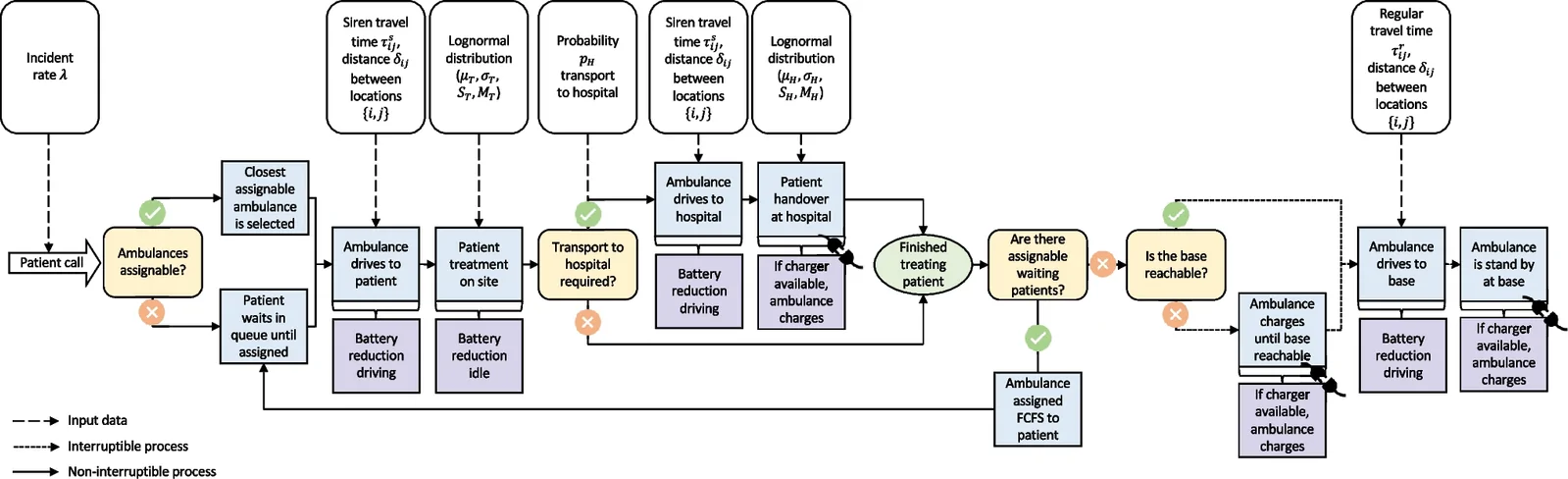
Overview of the process for the electric ambulance simulation

While other authors may have found a trade-off between model accuracy and computational complexity that they deem appropriate for a diesel fleet, we argue that any inaccuracies in this representation would have an even larger effect on response times in an electric fleet. Therefore, after carefully considering techniques such as stochastic programming, robust optimization and reinforcement learning, we concluded that these techniques would require far too much simplification of reality. As simulation allows the inherent randomness of the system and the dependence between vehicles to be modeled to a high extent, it is a more appropriate tool for the research question at hand.

Moreover, for practitioners, simulation is both familiar and trustworthy. Furthermore, it has been proven effective in communicating results with EMS providers [27]. This makes it a suitable tool to directly aid decision makers in their transition to electric vehicles, allowing them to assess the effects of electrification in their own region.

### Simulation model

We model the emergency response process by discrete-event simulation. Appendix A.1 explains the general emergency response process. Figures 1 and 2 show the simulation steps and the input data required for the diesel and the electric versions, respectively. The process for electric ambulances is different compared to diesel ambulances due to the involvement of batteries and charging. The input data is discussed in Section 3.4 and the simulation parameters are discussed in Section 3.5. We do not model triage and ambulance crew getting ready - an approach not uncommon in the ambulance planning literature [22, 47, 62].

The diesel ambulance simulation closely follows the emergency process of Fig. 14 of Appendix A.1. In the ELASPY code, the default is to use a spatial Poisson process that is implemented as follows. Patients arrive according to a Poisson process with parameter λ, i.e., we draw exponentially distributed interarrival times, and for each new arrival subsequently sample a discrete location with probability in proportion to an a-priori known demand distribution. There exists an alternative option to read in an Excel file with arrival times and locations, allowing the use of empirical data. After patient arrival, the same process as in Fig. 14 of Appendix A.1 is followed. It is assumed that the ambulance drives to the fastest-reachable hospital from a set *H*. Moreover, if there are waiting patients, the ambulance is assigned to a patient first-come-first-serve (FCFS); otherwise, the ambulance returns to its base. This return process is interruptible, meaning that the ambulance can be assigned to a newly arriving patient. In the case of a diesel ambulance, if necessary, the refueling is usually done by the crew just before they report themselves as idle. Since refueling is a quick and infrequent process, this is not included in the simulation. When a new patient arrives, the current locations of all available ambulances are used to select the closest ambulance. For ambulances that are returning to their base, we use linear interpolation between their origin and destination to determine their current position.

In the case of electric ambulances, we need to explicitly consider the battery utilization and charging processes. The other elements of the process are similar to those of the diesel simulation. At a workshop titled ‘Zero Emission Ambulance care’ organized by Axira [7] in 2022, the consensus between managers from different Dutch ambulance providers was that electric ambulances would and should not use public chargers. Instead, they envisioned that charging would happen entirely at hospitals and ambulance bases. Therefore, we assume that charging is only possible at ambulance bases and during patient handover at the hospital if there is an unoccupied charger present. If the ambulance cannot reach its base after the patient handover, we assume that the ambulance stays to charge at the hospital as long as necessary until it can reach its base. The number, type and speed of the chargers at each location are input data for the simulation (see Section 3.4). In this way, multiple charging scenarios can be considered.

The state of charge (SoC) of an ambulance reduces deterministically while driving. This is calculated by multiplying the traveled distance by the kWh/km energy consumption $B_D$. The SoC reduces at a different, also deterministic rate while the patient is treated on site, as the battery supplies electricity for the medical equipment on board. In this case, the battery reduction is calculated by multiplying the time spent on site by the kW energy consumption $B_I$.

An ambulance is considered “assignable" to a patient if (1) it is not assigned to another patient and (2) its SoC is sufficient to do the following:

- Drive from its current location to the patient, wait while the patient is treated on site, drive to the closest hospital with an emergency department (and in case no charger is present at the hospital, also drive back to its base from the hospital).
- Drive from its current location to the patient, treat the patient on site and drive back to its base.

The second option accounts for the case that the patient does not require transport to the hospital. Note that by using these requirements, the situation that an ambulance strands somewhere because of an insufficient SoC cannot arise, as then the ambulance would not have been assigned to the patient in the first place. The battery reduction that arises due to driving is known when the patient call arrives, since we assume deterministic energy consumption. However, beforehand it is not known how much time the ambulance will spend on site. Therefore, we assume that the ambulance must be able to spend the maximum treatment time $M_T$ on site for determining whether the ambulance is assignable or not. As the energy consumption of a stationary electric ambulance is relatively low, this modeling method does not have a large influence on the results. Note that the actual time spend on site is used to calculate the actual battery reduction later in the process.

We assume charging happens on a First-Come-First-Serve (FCFS) basis, which means that the ambulance that arrives first at the charger(s) charges first. If no chargers are available, the ambulances wait in a FCFS queue. If there are only regular or fast chargers at the location, then this is a single FCFS queue. If there are both regular and fast chargers, the ambulance starts charging at a fast charger if one is available; else at a regular charger, and if neither are free the ambulance joins the queue of the fast charger. At the bases, the ambulances charge until a full SoC or until they are assigned to a patient. During patient handovers at the hospital, the ambulances charge until a full SoC or until the patient handover has finished. In current practice, vehicles are already parked in a designated area outside the Emergency Department during patient handover. We envision the charging stations being installed at these locations. Consequently, charging can take place concurrently with the handover and does not introduce an additional out-of-service period, as the time required to connect the vehicle to the charger is assumed to be negligible. The ambulance re-enters the FCFS queue if the ambulance cannot reach its base and first has to charge at the hospital.

To validate whether the implemented simulation model correctly follows the processes as outlined in Figs. 1 and 2, simulation traces and outputs were carefully checked and analyzed for anomalies in each component of the simulation. Moreover, implemented unit and regression tests further safeguard the quality of the model. Unit tests check whether the behavior of small parts of the code is as expected, and regression tests check whether the results of new code versions are equal to those of previous code versions. The diesel simulation runs fastest as there are fewer events to consider than in the case of electric ambulances due to the charging processes. A single run that models a 12-hour period with diesel ambulances takes on average 0.21 seconds, whereas a similar run with electric ambulances takes on average 0.29 seconds on a 2.3 GHz 8-Core Intel Core i9 with 16GB of RAM laptop. In general, the running time increases for busier systems. The code and data are bundled into the package Electric Ambulance Simulator Python (ELASPY), which is publicly available [1]. Documentation, explanations and set-up guidelines can be found on  1 and 2 [2].

### Case study

We consider the province of Utrecht, the Netherlands, for a case study. The province is a relatively compact and flat geographical area, stretching approximately 60 kilometers east–west, with a population of around 1.4 million inhabitants distributed over 26 municipalities. The region is characterized by the absence of significant elevation differences and a moderate climate, which ensures relatively stable travel conditions year-round. Ambulance care is provided by a single regional provider RAVU [51]. RAVU served 97,607 patients in the year 2021 [4, p.63]. Out of those patients, 67,855 (69.5%) were urgent (triaged as priority A). RAVU distinguishes between advanced life support (ALS) and basic life support (BLS) vehicles; however, in this study we restrict our analysis to ALS units.

Utrecht’s emergency call center is integrated into a broader dispatch system that serves multiple regions beyond Utrecht. Call takers use a computerized triage system to decide whether an ambulance is needed, as well as the required vehicle type (ALS or BLS). Subsequently, information regarding the incident location and urgency is forwarded to a separate team of dispatchers, who decide which vehicle to send (which in case of urgent calls would be the closest idle vehicle).

**Fig. 3:**
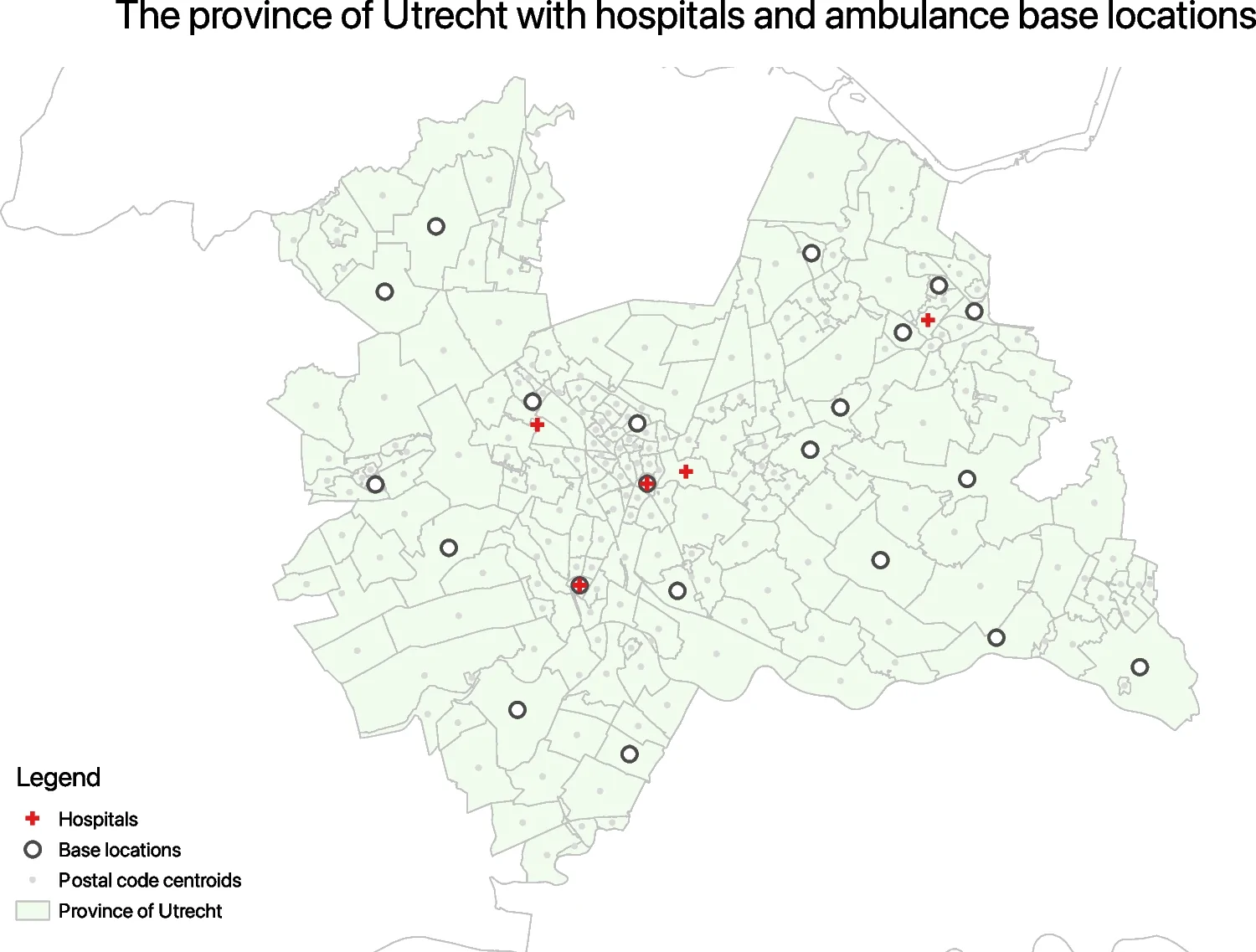
The province of Utrecht with the centroids of the postal codes, hospitals with an emergency department and RAVU base locations

The EMS system in Utrecht shares some properties with the rest of the Netherlands. The national target for urgent calls (so-called A1 calls) is that 95% of the calls are reached within 15 minutes [36, p.16]. Practitioners often report the 95% quantile of the response times as proxy for this service-level agreement [4]. Unlike in countries such as the United Kingdom, Dutch hospitals do not experience systematic ambulance queues outside emergency departments, hence there is no need to account for idle time upon arrival. An additional operational feature is that ambulance repositioning–actively relocating idle ambulances to improve spatial coverage–was popularized in the Netherlands around 2015, sometimes supported by academic work and software tools (see, e.g., Van Buuren et al. [63]). However, in recent years a noticeable shift away from this practice has been observed, driven by concerns over employee dissatisfaction with frequent relocations and managerial worries about staffing shortages (director of multiple Dutch ambulance services, personal communication, May 2022).

Utrecht’s EMS provider operates from 21 different base locations (locations provided in personal communication with RAVU, 2021). The region has five hospitals with an emergency department that are used to transport patients to when needed. The bases and hospitals are shown in Fig. 3, with hospital locations derived from [67].

**Table 1:** Input data for the simulation for the region of Utrecht

Symbol	Definition	Source
*V*	Demand locations in Utrecht discretized to 4-digit postal codes in 2021	Based on CBS data [13, 14]
$d_i$	The demand for location *i* based on the fraction of inhabitants in 2021	Based on CBS data [14]
W⊂V	Base locations as existing per 2021	Based on personal communication with RAVU
*W*(*A*)	Ambulance assignment to base locations	Based on RAVU data and MEXCLP
H⊂V	The hospitals that have an emergency department within the province of Utrecht in 2021	Based on data VZinfo [67]
$\tau ^r_{ij}$	Regular driving time between locations *i* and *j*	Derived from OpenStreetMap [49]
$\tau ^s_{ij}$	Siren driving times obtained by calculating $0.95 \times \tau ^r_{ij}$	n/a
$\delta _{ij}$	Distance between locations *i* and *j*	Derived from OpenStreetMap [49]

### Input data

Table 1 presents the input data used in this research. The province Utrecht is divided into 231 different 4-digit postal codes, constituting our demand nodes, which we position in their geographical centroid, and denote as set *V* (see Fig. 3). These postal codes are based on data provided by Centraal Bureau voor de Statistiek [13, 14]. For the demand $d_{i}$ in node i∈V, we take the population fraction in that node [14]. In reality, the ambulance demand could differ from the population fraction; however, we consider this a reasonable proxy and it has the benefit that we are allowed to share the data. We take the set of hospitals *H* and base locations *W* as described in Section 3.3 and Fig. 3. Each ambulance is assigned to one of these bases as their home location, and this choice is determined by mathematical optimization. Given *A* ambulances, we solve the Maximum Expected Coverage Location Problem (MEXCLP) [20] to determine at which base to position each ambulance, leading to base assignment *W*(*A*). Section 3.5 contains more details on this algorithm. .

The regular driving times $\tau ^r_{ij}$ and distances $\delta _{ij}$ between any two nodes i,j∈V are derived from OpenStreetMap [49]. These are driving times for regular cars driving at maximum allowable speed. We assume these are also realistic for ambulances when they are driving back to their base. To reflect that ambulances with urgency are faster, we model the driving times of lights and siren trips to be 5% lower. For all trips towards the patient and for transport to the hospital, we assume lights and sirens are used.

The number, type and speed of chargers at each base and hospital also forms input of the simulation. The user can assign regular (R) and fast (F) chargers to each base (B) and hospital (H) separately. In this paper, we use the encoding RB#_RH#_FB#_FH# to specify the number (#) and type (R, F) of charging stations at each base and hospital. If a term is omitted, then it means that no charging stations of that type are available at the location. In our experiments, the number of chargers is equal for all locations of the same type (i.e., base or hospital), but the software is flexible enough to allow this number to vary per location. Our regular chargers have a power output of 11 kW and fast chargers supply 50 kW, but other values can be specified by the user. These are values that are also used in the literature [10, 18].

Table 2 presents the charging station scenarios that are considered in our experiments. Scenario RB1 is the most restricted scenario with only one regular charger per base. The other scenarios are realistic variations that can be used to gain insight into how many and which type of chargers are effective. During the exploratory analysis phase, we considered more charging scenarios, for example RB1_FB1, RB50_RH50 and RB50_FH50 (the last two reflecting “unlimited" charging scenarios), but we found that the charging scenarios in Table 2 were most interesting for our case study. The scenarios where no charging stations are present at hospitals are interesting to consider, since then the ambulance care providers have full control over where, how many and which charging stations are placed. Furthermore, fast chargers may require changes to the grid and are typically more expensive than regular chargers, which makes it especially relevant to determine whether regular chargers are sufficient for running operations.

**Table 2:** The considered charging scenarios

		Chargers at hospitals?	
Fast chargers?		Yes	No
	**Yes**	FB1_FH1	FB1
		FB1_RH1	
		RB1_FH1	
	**No**	RB1_RH1	RB2
			RB1

### Simulation parameters

Besides input data, the simulation also requires several input parameters. These are discussed in this section. Table 3 presents the simulation input parameters.

The incident rate λ for the Poisson patient arrival process is based on data from Ambulancezorg Nederland [4, p.63]. This region’s 67,855 annual urgent trips, as described in Section 3.3, lead us to define an average time between incidents of 7.75 minutes.

**Table 3:** Parameters of the simulation for the region of Utrecht

Parameter	Value	Definition	Source
λ	1/7.75 min	Poisson rate of the arriving patient process	Deduced from Sectorkompas Ambulancezorg 2021 [4]
$\mu _{T}$	3.61 min	Mean of the underlying normal distribution of the on-site treatment duration	Empirical data
$\sigma _{T}$	0.38 min	Standard deviation of the underlying normal distribution of the on-site treatment duration	Empirical data
$S_{T}$	-10.01	Shift parameter of the on-site treatment duration	Empirical data
$M_{T}$	88 min	Maximum on-site treatment duration	Empirical data
$\mu _{H}$	3.58 min	Mean of the underlying normal distribution of the handover time at hospital	Empirical data
$\sigma _{H}$	0.39 min	Standard deviation of the underlying normal distribution of the handover time at hospital	Empirical data
$S_{H}$	-8.25	Shift parameter of the handover time at hospital	Empirical data
$M_{H}$	88 min	Maximum handover time at hospital	Empirical data
$p_{H}$	0.63	Probability that a patient requires transportation to the hospital	Deduced from Sectorkompas Ambulancezorg 2021 [4].
*X*	5000	Number of simulation runs	Value based on experiment (see Appendix A.2)
*A*	20	Number of ambulances	Realistic number to cover observed demand in [4]
*B*	150 kWh	Battery capacity of an electric ambulance	Value estimated by industry experts
$B_D$	0.4 kWh/km	Energy consumption during driving	Value provided by industry experts
$B_I$	5 kW	Energy consumption when stationary (idling)	Value provided by industry experts
*T*	720 min	Time horizon during which patients arrive	n/a
$t_w$	1 min	Time between intervals to check for waiting patients that can be helped by charging ambulances	n/a
ϕ	-	Seed value used for reproducibility	Value depends on the experiment

Distributions for on-site treatment times and hospital handover times were estimated by using empirical data. The data contained considerable outliers, which we believe originated from human errors in the recording of the times. While this may be different for datasets from other systems, we deemed it appropriate to ignore values above the 99%-quantile, which we denote by $M_T$ and $M_H$. Coincidentally, having some maximum on these times proved useful for the simulation (see Section 3.2). We then used Maximum Likelihood estimation to fit several well-known distributions to the data and chose the best fit. Both data sets turned out to be best reflected by the lognormal distribution, which is a common distribution for service times [11, 17]. The on-site treatment time follows a lognormal distribution with parameters $\mu _T$, $\sigma _T$ shifted by $S_T$. The hospital handover time follows a lognormal distribution with parameters $\mu _H$, $\sigma _H$ shifted by $S_H$. Values lower than zero or higher than $M_T$ or $M_H$ are regenerated until a value between these boundaries is returned.

In some cases, patients only need to be treated on site and do not need to be transported to a hospital. We estimate the probability $p_{H}$ that a patient has to go to a hospital by dividing the number of transported patients (denoted as declarable trips in Ambulancezorg Nederland [4, p.68]) by the total number of trips. This results in a probability $p_H$ of 0.63.

The simulator can deal with stopping criteria based on a number of patients *N* or a time interval of *T* minutes. Unless mentioned otherwise, the experiments reported in this paper consist of runs that represent 12 hours (i.e., T=720, *N* not relevant). For each run, the simulation starts with an empty system and electric ambulances start with full batteries. We consider this setup realistic when the beginning of the time window represents the early morning, since ambulances in the Netherlands are idle enough during the night to recharge to full capacity.

A single simulation run is insufficient to draw conclusions due to the stochasticity of the system. Therefore, we run the simulation *X* times and take the mean over the resulting *X* values of a performance measure and report this in our results. There is a trade-off in the choice of *X*: a large value results in high running times, whereas a low value in low accuracy. Unless mentioned otherwise, we take X=5000, based on an experiment explained in Appendix A.2.

We fit the number of ambulances *A* so that our simulated diesel response times approximate those achieved in reality by the RAVU [4, p.78]. For this, we use mathematical optimization and publicly available data on the response times of the ambulances in the province of Utrecht. We use an iterative optimization process: we start by running *X* runs of the simulation for *Y* ambulances and note down the mean 95% quantile of the response time over all runs. We compare this to the actual reported 95% quantile of the response time found in [4, p.78]. If the simulated mean 95% quantile is lower than the actual quantity, then we repeat the process with Y-1 ambulances, and if it is higher, we repeat the process with Y+1 ambulances. We choose to fit on the 95%-quantile of the response time, as this quantity is the main performance measure for ambulance care providers in the Netherlands. We run the simulations with diesel ambulances, since we want to fit the number of ambulances to the current situation.

To determine the distribution of the *Y* vehicles over the bases *W*, we also use mathematical optimization: we solve the Maximum Expected Coverage Location Problem (MEXCLP) [20]. MEXCLP places ambulances as to maximize on-time arrivals and requires an input parameter called the busy fraction, denoted *q*. This parameter should be interpreted as the probability that any ambulance is busy at any time. As input the model further requires demand nodes *V* with demand $d_i$ (i∈V) and travel times (with lights and siren) $\tau _{ij}^s$, i,j∈V.

For completeness, we next reiterate the MEXCLP model. Consider a demand node i∈V that is reachable within the threshold time by *k* ambulances, then the expected covered demand of this node is $E_k = d_i (1-q^k)$. Consequently, the marginal contribution of the *k*th ambulance to this expected value is $E_k - E_{k-1} = d_i (1-q)q^{k-1}$. MEXCLP introduces a binary variable $y_{ik}$ that is equal to 1 if and only if vertex i∈V is within range of at least *k* ambulances. The variables $x_j$ represent the number of vehicles at base j∈W. Define $W_i$ as the set of bases from which demand point *i* can be reached within the threshold time (which can be precomputed), then the MEXCLP model is formulated as follows:

$$\begin{aligned}&\text {Maximize }  &   \sum _{i \in V} \sum _{k=1}^{Y} d_i (1-q)q^{k-1} y_{ik} \\&\text {subject to }  &   \sum _{j \in W_i} x_j \ge \sum _{k=1}^{Y} y_{ik},  &   i \in V, \\  &    &\sum _{j \in W} x_j \le Y, \\  &    &x_j \in \mathrm {\mathbb {N}},  &   \!\!\!\!\!\!\!\! j \in W, \\  &    &y_{ik} \in \{ 0,1 \},  &   \!\!\!\!\!\!\!\! i \in V , k \!=\! 1,\ldots ,Y. \end{aligned}$$

There is no need to add the extra constraint $y_{ih} \le y_{ik}$ for h≤k. This will always hold for an optimal solution, since $E_k - E_{k-1}$ is decreasing in *k*.

In our implementation we take a value of q=0.6, as suggested by the Dutch Institute for Public Health and the Environment [36, p.16] (in Dutch: ‘bezettingsgraad’). We set the threshold for the driving time to 12 minutes (the national target for A1 calls, see Section 3.3) minus 3 minutes for call handling and chute time [68].

The pseudo-code for iteratively fitting the number of ambulances is provided in Algorithm 1. We use $r_{actual} =$ 14:21 minutes^1^ and X=5000 runs. We run algorithm 1 and we find that A=20 yields a mean 95% quantile of 14:20 minutes.

**Algorithm 1:**
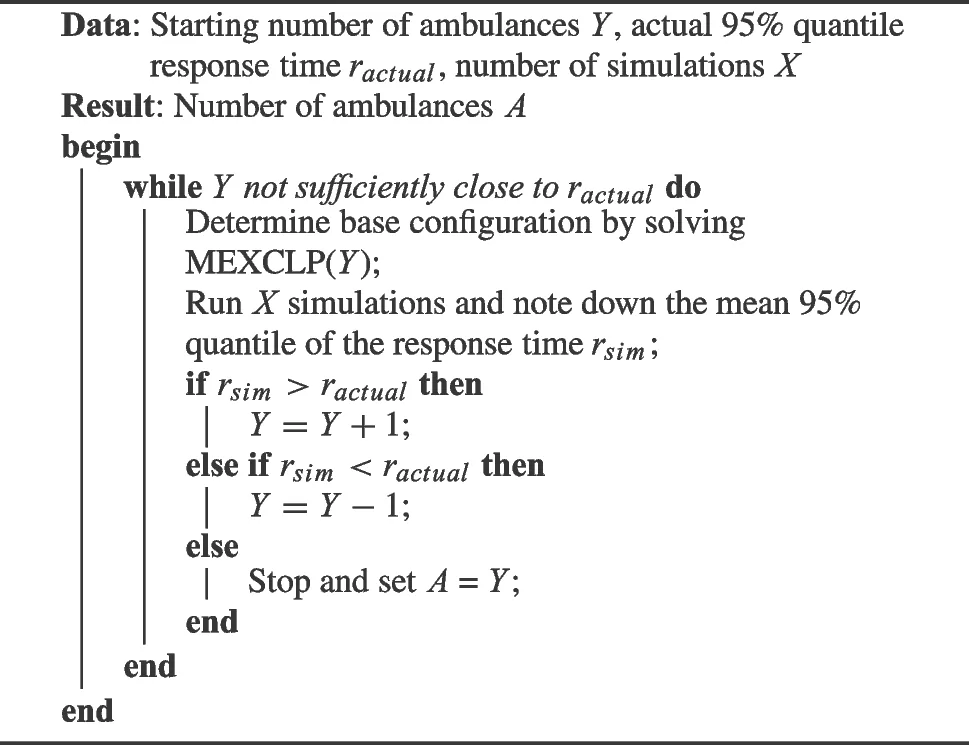
Pseudo-code for iteratively fitting the number of ambulances.

The battery capacity of an electric ambulance is denoted by *B*, and we set this at 150 kWh, as estimated by our contacts at Axira and Mercedes-Benz Netherlands. There may be instances where an electric ambulance, after assisting a patient, is unable to immediately assist a new patient due to insufficient battery capacity. Therefore, with the electric version, the simulator checks every $t_w$ minutes whether the ambulances that are charging at that moment can help any of the waiting patients in a FCFS manner.

The simulator can both use historical data and data generated by a random number generator for the interarrival times, on-site service times, drop-off times, call locations and the need for hospital transport per patient. If the random number generator is used, the user can specify a seed, ϕ, to ensure reproducibility. The experiments in this paper are based on data generated with a random number generator. To decrease the interdependence of different experiments, the seed can vary between experiments.

The user can easily specify the simulation parameters through the main interface, which makes the simulator both flexible and accessible. To increase user-friendliness, we included a user input validator, which checks whether the specified input is valid or not.

**Fig. 4:**
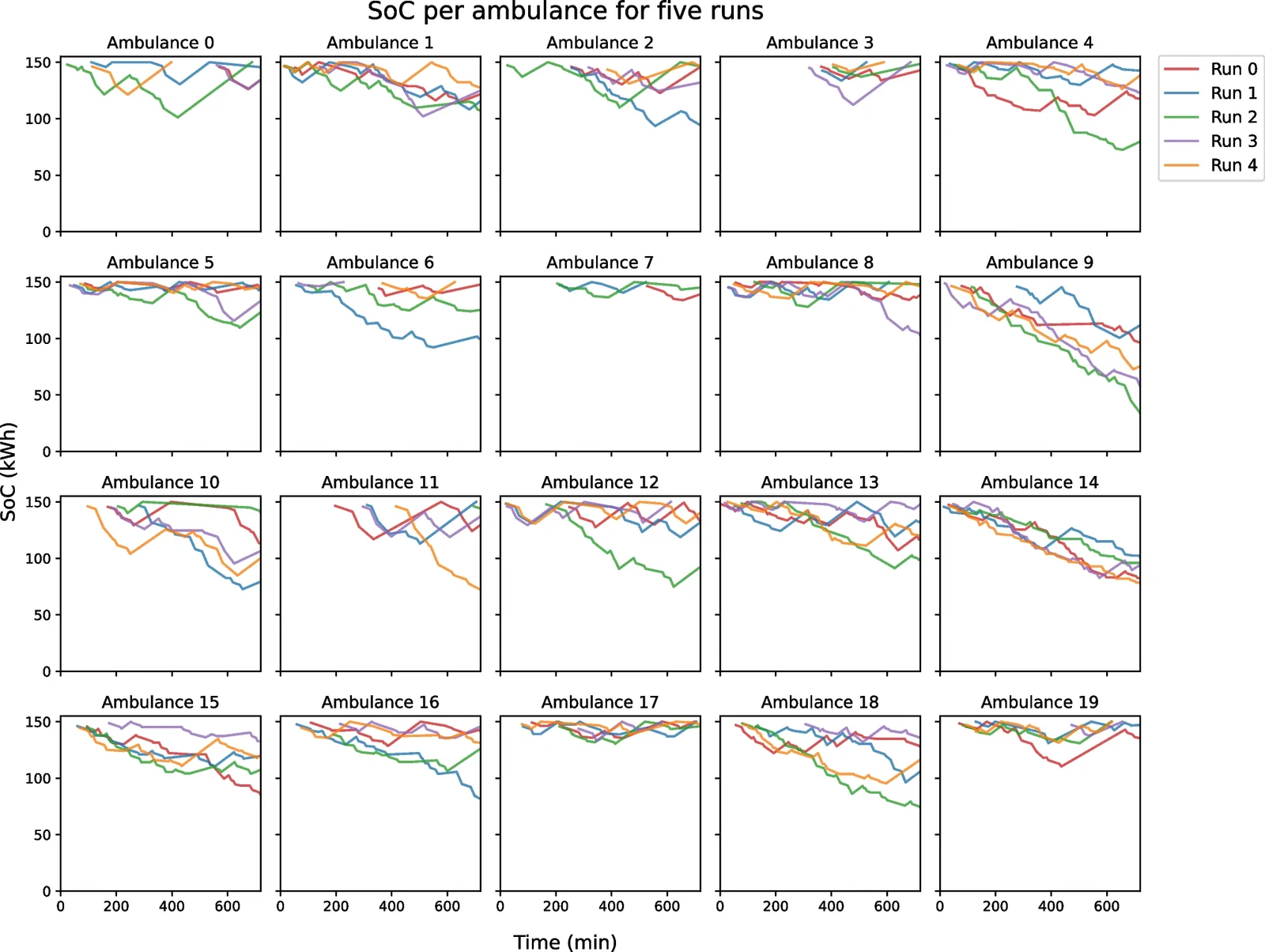
The state of charge (SoC) of all ambulances of five simulation runs during the 12-hour period

### Output data

The output of the simulator consists of a variety of data. First of all, important performance measures such as the mean response time, the 95%-empirical quantile of the response time and the busy fraction are provided per run, but also as 95%-confidence intervals and means for all the runs aggregated. Detailed data on patient and ambulance processes is exported as tables in csv files, which can also be used to analyze differences between multiple scenarios. This data can also be used to verify that the simulation is working as expected. For more information about the possible output, we refer to the ELASPY website [2].

## Results

In this section, we discuss the numerical experiments that we performed with the developed simulator to gain insights into which factors are important for the transition to electric ambulances. We start by running simulations that compare the performance of diesel and electric ambulances with the parameters provided in Table 3. We refer to this setting as the “expected situation" since the parameters are based on the prognoses of experts. Next, we perform sensitivity analyses to gain more insights in the robustness and behavior of the system under varying conditions in Section 4.2. In Section 4.3, we use ELASPY to conduct a simulation-based optimization study to optimize charger allocation. In Section 4.4, we derive managerial insights from our numerical experiments. We base our findings on the mean and 95%-quantile of the response times. Especially the 95%-quantile is an important KPI in the ambulance sector in the Netherlands [21], which is considered a direct proxy of the national service-level agreement that 95% of the urgent calls are reached within 15 minutes.

### Expected situation

We simulate and compare fleets of diesel and electric ambulances using the parameters of Table 3. We consider all charging scenarios in Table 2. Interestingly, there is not much difference between the mean response times and 95% empirical quantiles (referred to as 95%-QRT in the following) for all electric scenarios compared to those of the diesel fleet (Fig. 16 in Appendix B.1). This result can be explained by considering the SoC of all ambulances during the 12-hour period of the most limited charging scenario, RB1. Figure 4 shows that none of the ambulances reach a low SoC throughout the day. Therefore, in terms of response times, the performance of an electric fleet is similar to that of a diesel fleet.

Observing no issues within a 12-hour horizon raises the question: After how many hours of continuous operation would electric ambulances run into problems? An additional experiment shows that under the most limited charging scenario, RB1, the time until the first vehicle reaches a SoC below 20% was approximately 17 hours (average over X=1000 runs, T=2500).

Besides reporting these ambulance-specific performance measures, it is also interesting to discuss some statistics on battery usage and charging times. Table 4 presents some statistics for the RB1 scenario. The mean is taken over all X=5000 simulation runs to arrive at the statistic. The statistics suggest that charging sessions are usually short, except for the charging sessions at the end of the day. The mean battery increase per charging session is larger than the mean battery decrease per ambulance-action (driving or idling), which supports the result that the system operates adequately under the RB1 scenario.

Based on the total distance driven by all ambulances, we can estimate the resulting CO2 reduction. According to van der Hoogt [65], electrifying ambulances reduces emissions by an estimated 328 g CO2e/km (wheel-to-wheel, assuming 87% charging efficiency) compared to diesel. Using the statistic from Table 4, we estimate the total CO2 reduction for urgent ambulance trips within Utrecht to be 705.25 kg CO2e for a single day of operation, resulting in a rough estimate of 257 tonnes of CO2e reduction per year.

**Table 4:** Statistics on battery usage and charging times for the RB1 scenario under the expected situation

Statistic	Value
Total driven kilometers all ambulances	2150.17
Mean time of a charging session (min)	82.49
Median time of a charging session (min)	45.19
Mean total charging time single ambulance (min)	305.18
Mean battery increase (kWh)	15.12
Mean battery decrease (kWh)	3.21

The experiments of this section suggest that transitioning to electric ambulances under the parameters as expected by experts would not impact the response times significantly, even not under the most limited charging scenario, RB1. However, as most parameters are still uncertain, it is interesting to determine what the influence is if they change. Moreover, ambulance care providers operate under high service levels since lives are at stake. This means that these should also be met in exceptional cases. In other words, robustness of the system is of utmost importance. The following section will therefore consider multiple scenarios under varying circumstances.

### Sensitivity analysis

#### Increasing the busyness of the system

In these experiments, we consider what happens if the system gets busier. We model this by increasing the arrival rate λ. This means that the interarrival time between patients becomes smaller, increasing the number of patients that arrive during the 12-hour period. We evaluate $\lambda \in \{\frac{1}{6}, \frac{1}{5}, \frac{1}{4}\}$. Increasing the arrival rate even more is illogical, as the system then also becomes too busy for a diesel fleet of the same size.

**Fig. 5:**
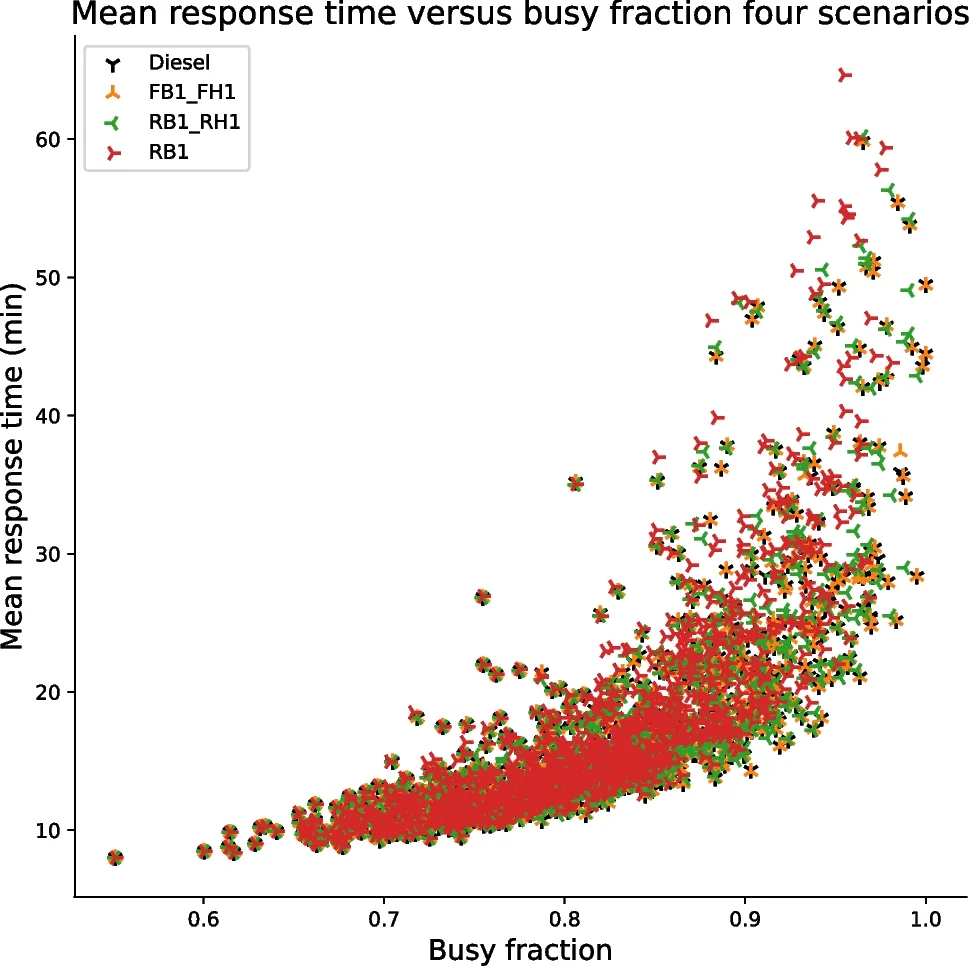
The mean response time versus the busy fraction for a diesel fleet and three charging scenarios for an electric fleet for 1000 simulation runs each. Notice that runs with identical results form (semi-)circles

Figure 5 presents the mean response time versus the busy fraction for the three most interesting charging scenarios and the diesel case. For calculating the busy fraction, we use a warm-up and cool-down period to not incorporate data from an almost empty system into the busy fraction. The plot suggests that scenario RB1 is sufficient in systems with a busy fraction lower than 0.85. However, once the busy fraction of the system becomes higher than 0.85, the performance start to deviate under the RB1 scenario. The performance with charging scenario FB1_FH1 does seem to be very close to the diesel performance even in very busy systems. This is also supported by Fig. 17 in Appendix B.2 that shows the two performance measures for all charging scenarios for λ=1/4. As expected, especially in a (very) busy system sufficient charging capacity should be present to be able to reach a performance comparable to the diesel fleet. If this capacity is not available, the performance starts to deteriorate significantly, as can be seen by a 95%-QRT of approximately 200 minutes for scenarios RB2 and RB1 in Fig. 17, for example.

#### Reduced battery capacity

The results of the previous experiments suggest that charging scenario RB1 is sufficient under the expected situation. However, what happens to the performance if the battery capacity is reduced? This is relevant in at least three situations. First, it might be the case that only batteries with less than 150 kWh capacity can be purchased at the start of the transition. Second, it is known that cold weather influences battery capacity [76, 78] and lastly, the capacity of batteries typically reduces over time [9].

We investigate for which battery capacity the performance starts to differ significantly from the diesel fleet. That is, we seek how low the capacity can be until the confidence interval^2^ no longer overlaps with the confidence interval of the diesel fleet. We run this experiment using the parameters of Table 3 and the most limited charging scenario, RB1. We use binary search with X=5000 to find the value for battery capacity *B*. We find that, as long as the battery capacity is above 115 kWh, the confidence intervals of the 95%-QRT of the electric and diesel fleets overlap. In other words, with battery capacities below or equal to 115 kWh, we can be sure to expect performance to differ from the diesel fleet. If RAVU were to purchase such vehicles, we would certainly advise them that charging scenario RB1 is insufficient.

#### Reduced battery capacity and increased busyness

To put the system under more strain, we consider what happens if vehicles use batteries of 115 kWh and the system becomes busier. We consider $\lambda \in \{\frac{1}{6}, \frac{1}{5}, \frac{1}{4}\}$. Our experiments suggest that the performance of the electric ambulances in terms of response time starts to decrease, although it is still possible to reach a performance similar to that of a diesel fleet if sufficient charging stations are present.

**Fig. 6:**
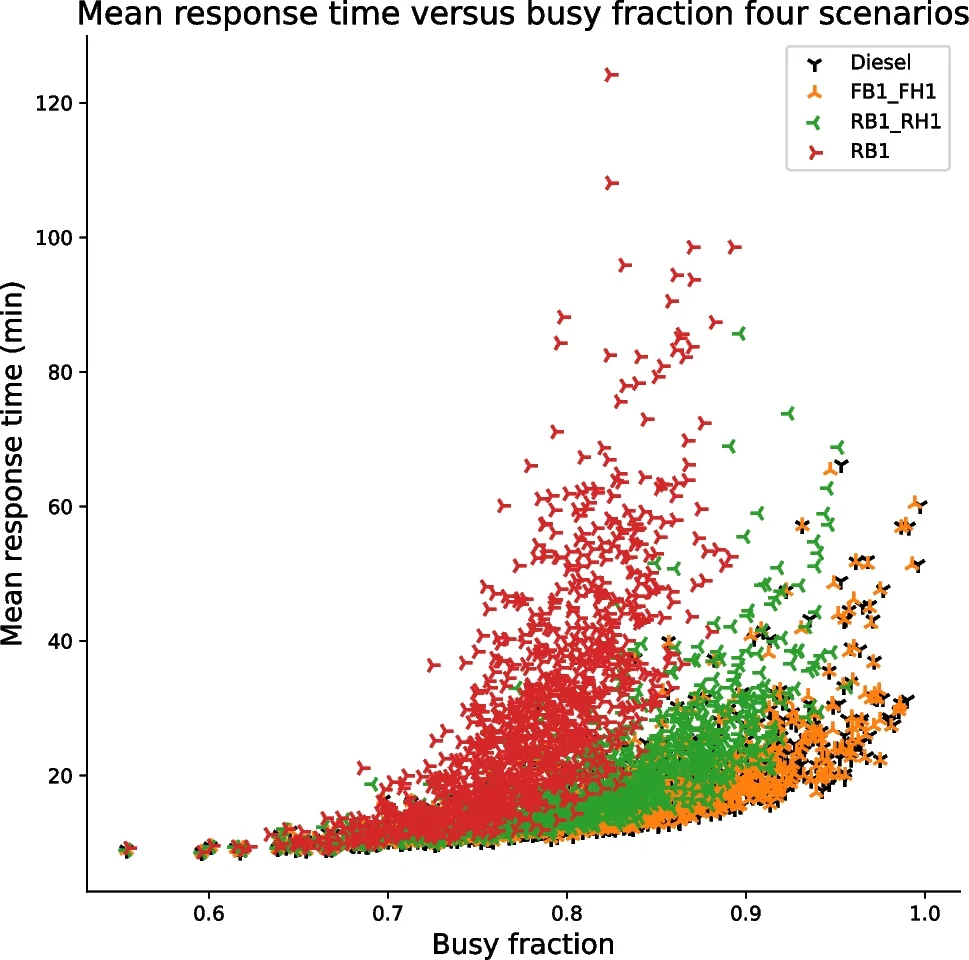
The mean response time versus the busy fraction for a diesel fleet and three charging scenarios for an electric fleet for 1000 simulation runs each

**Fig. 7:**
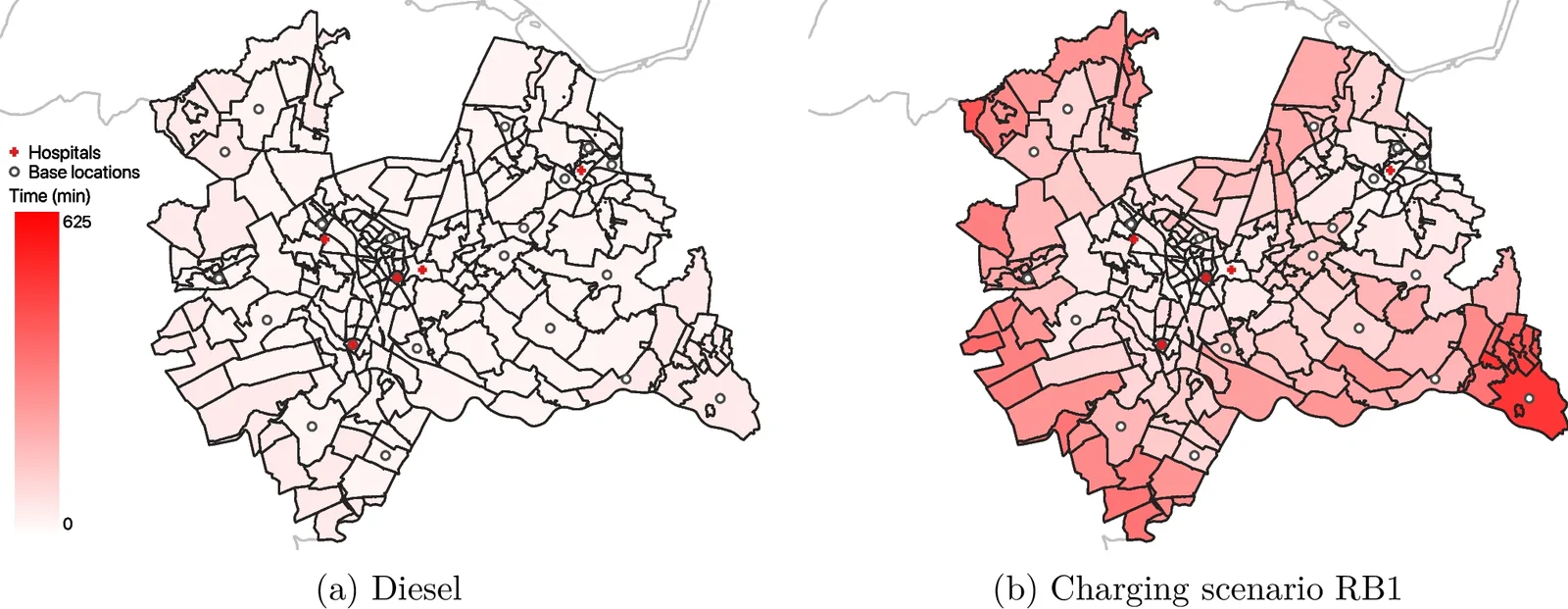
The 95% empirical quantile of the response times per postal code of the province of Utrecht

Figure 6 presents the mean response time versus the busy fraction for the diesel case and three charging scenarios. Charging scenario RB1 is clearly not sufficient anymore and probably life-threatening situations arise, since mean response times can be as long as 120 minutes even for busy fractions around 0.8. Adding a regular charger at the hospital has a profound positive influence (RB1_RH1). Moreover, charging scenario FB1_FH1 seems to result in a performance very similar to that of a diesel fleet. Figure 18 in Appendix B.3 presents the results for all scenarios and λ=1/4. Figure 7 gives insight into how the 95%-QRT is distributed over the province of Utrecht for the diesel and RB1 scenarios with λ=1/4 and B=115. Although the 95%-QRT are quite extreme (with a maximum of 614 minutes) and therefore not very realistic, the two plots do show where problems arise in the region. High response times are observed mainly in the outskirts and in the south-east part of the region, for both scenarios.

Shapiro [56] mentions a desired requirement for ambulances in England to have a range of 160 miles, i.e., approximately 257 kilometers. Given the expected energy consumption during driving (0.4 kWh/km), this would be equal to a battery of approximately 105 kWh. These experiments suggest that already life-threatening situations can arise if batteries of 115 kWh are used and not sufficient charging stations are available.

#### Mixed fleets

Some ambulance providers may be interested in operating a hybrid fleet consisting of both diesel and electric ambulances. This may arise, for example, during a transition towards full electrification or to improve operational performance during periods of high demand. To illustrate this, we consider the worst-case scenario from the “reduced battery capacity and increased busyness” experiment above and investigate the effect of replacing half of the electric fleet with diesel ambulances. Specifically, we consider a 50/50 hybrid fleet (10 electric and 10 diesel ambulances) under λ=1/4, B=115, and charging scenario RB1. The 10 ambulances with the lowest mean battery levels in the previous experiment are selected as the diesel vehicles. The simulation is then run for X=5000 replications.

**Fig. 8:**
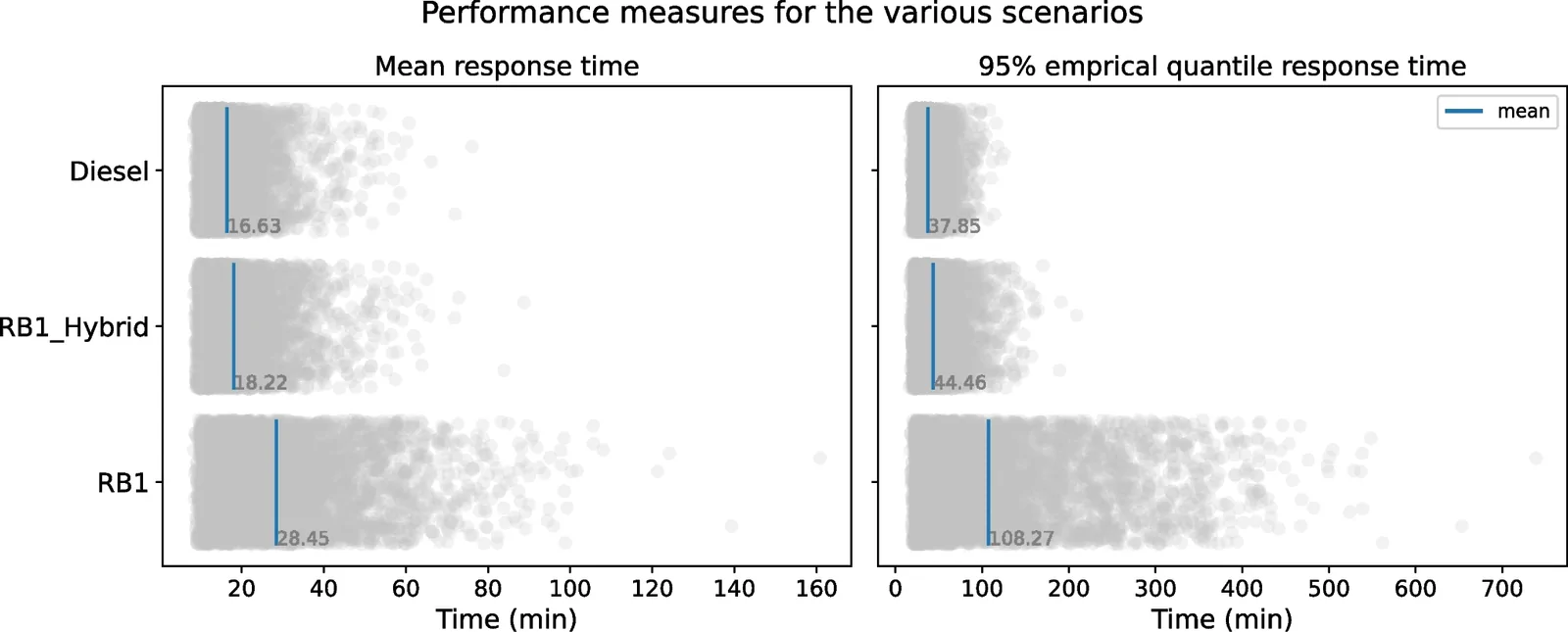
The mean and 95% empirical quantile of the response times of all scenarios for λ=1/4, B=115, X=5000 and a 50/50 hybrid fleet

Figure 8 presents the results for this scenario and compares them with those of a fully diesel and a fully electric fleet. The performance of the hybrid fleet is substantially closer to that of the fully diesel fleet. This suggests that hybrid fleets may provide an attractive intermediate solution during the transition to full electrification, mitigating many of the operational drawbacks of a fully electric fleet while still delivering part of the environmental benefits.

#### Increased Energy Consumption

The energy consumption during driving and treating the patient on site may also differ from the expected values. Therefore, we also consider what happens if the energy consumption increases. This can be, for example, interesting in hilly terrain where energy consumption is generally higher [58], varying weather conditions [76, 78] or if it turns out that the energy consumption of the ambulances is in general higher than expected. We use the parameters of Table 3, but increase the energy consumption during driving and idling by 20% to 140% in increments of 20%. We take X=500 and charging scenario RB1.

**Fig. 9:**
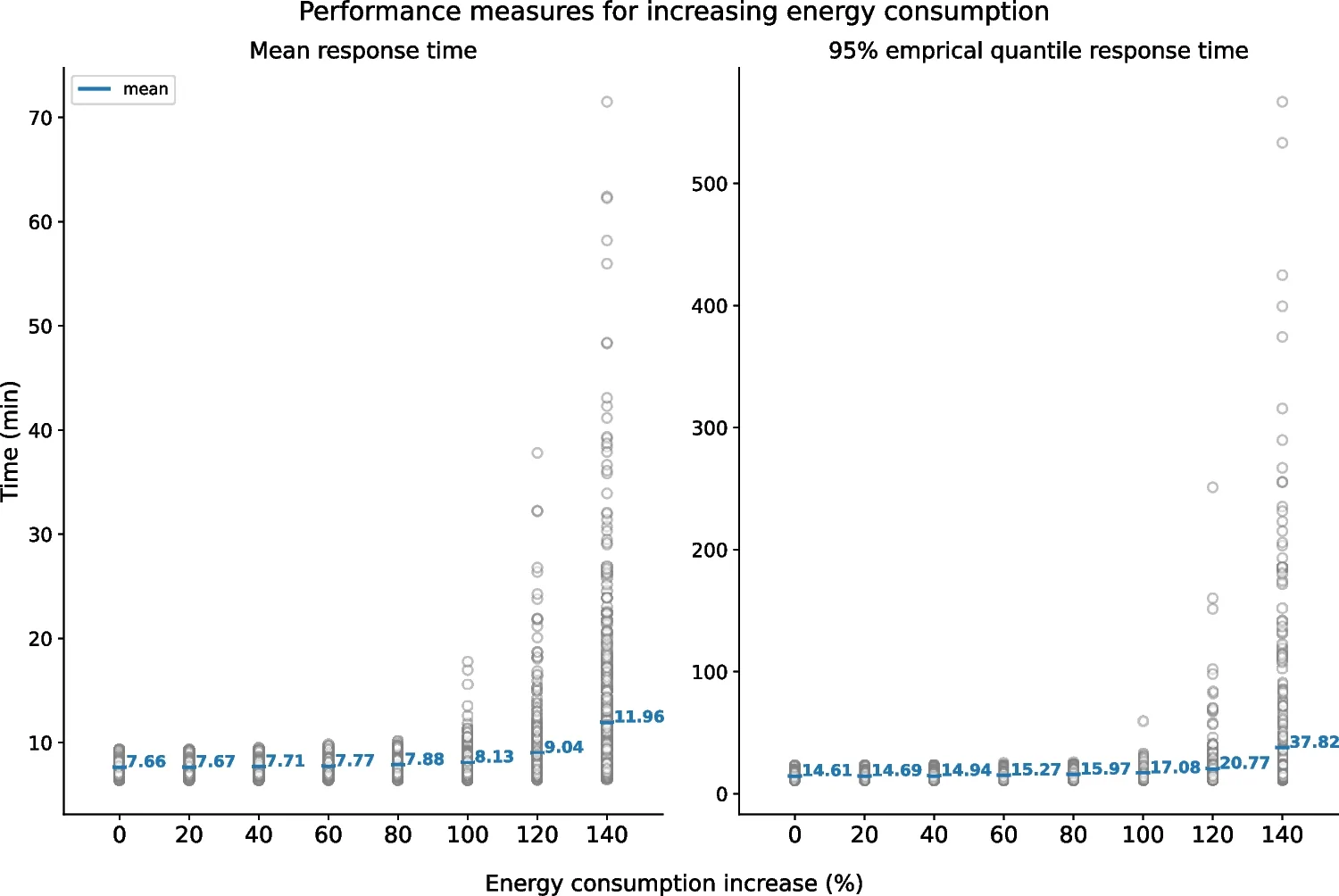
The mean and 95% empirical quantile of the response times for increasing energy consumption and scenario RB1

Figure 9 shows what happens to the mean response time and 95%-QRT for higher energy consumption. If energy consumption is increased by more than 60%, the confidence intervals of the performance measures are non-overlapping, indicating a significant difference. Especially a doubling of the energy consumption or more (>100%) increases the response times to unreasonable levels, such as a 95%-QRT of 200 minutes.

#### Non-linear Energy Consumption

In the previous experiments, the energy consumption during driving had a linear relationship with the trip length. In this experiment, we evaluate what happens if this relationship is non-linear. The electricity usage per kilometer is now, instead of $B_D$, denoted as $B_{D}(x)$, a (deterministic) function of the trip length x.

**Fig. 10:**
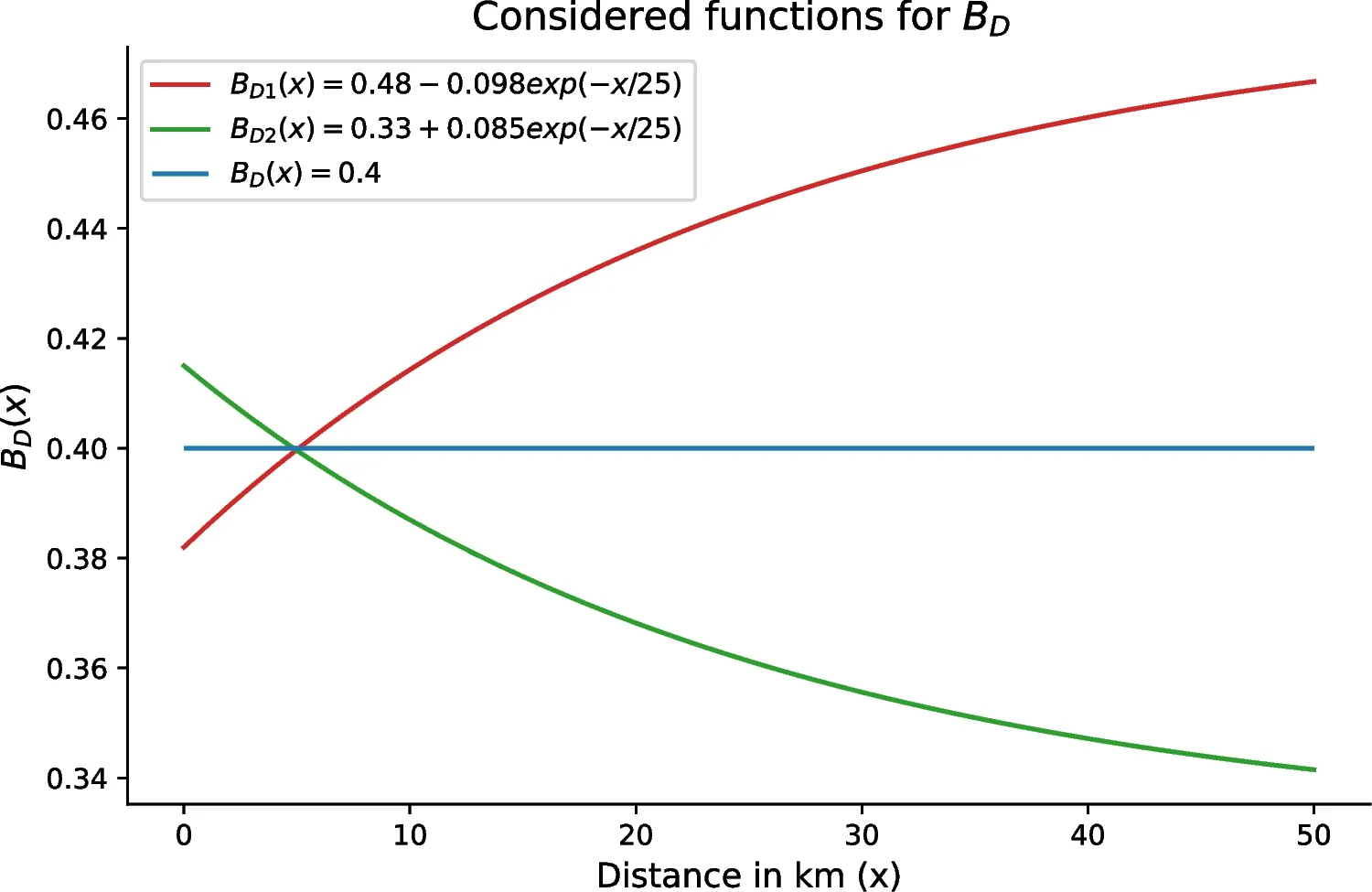
The two considered functions for the energy consumption during driving factor $B_D$, expressed in kWh/km. The two functions are shown alongside the constant value $B_D$ that was used earlier

We consider two functions: $B_{D1}$ and $B_{D2}$. With $B_{D1}$ the discharge per km grows with trip length - this reflects that longer trips are more likely to use the highway, and therefore to reach the very high speeds that require a lot of discharge. With $B_{D2}$ the discharge per km decreases with trip length - this reflects that short trips tend to face a lot of overhead because frequent turns and parking take up a relatively large portion of the trip. These functions are shown in Fig. 10, and were calibrated such that for a 5-km trip -roughly average for Utrecht- both functions come down to a discharge equal to the constant discharge of $B_D$ of 0.4 kWh/km from before.

**Fig. 11:**
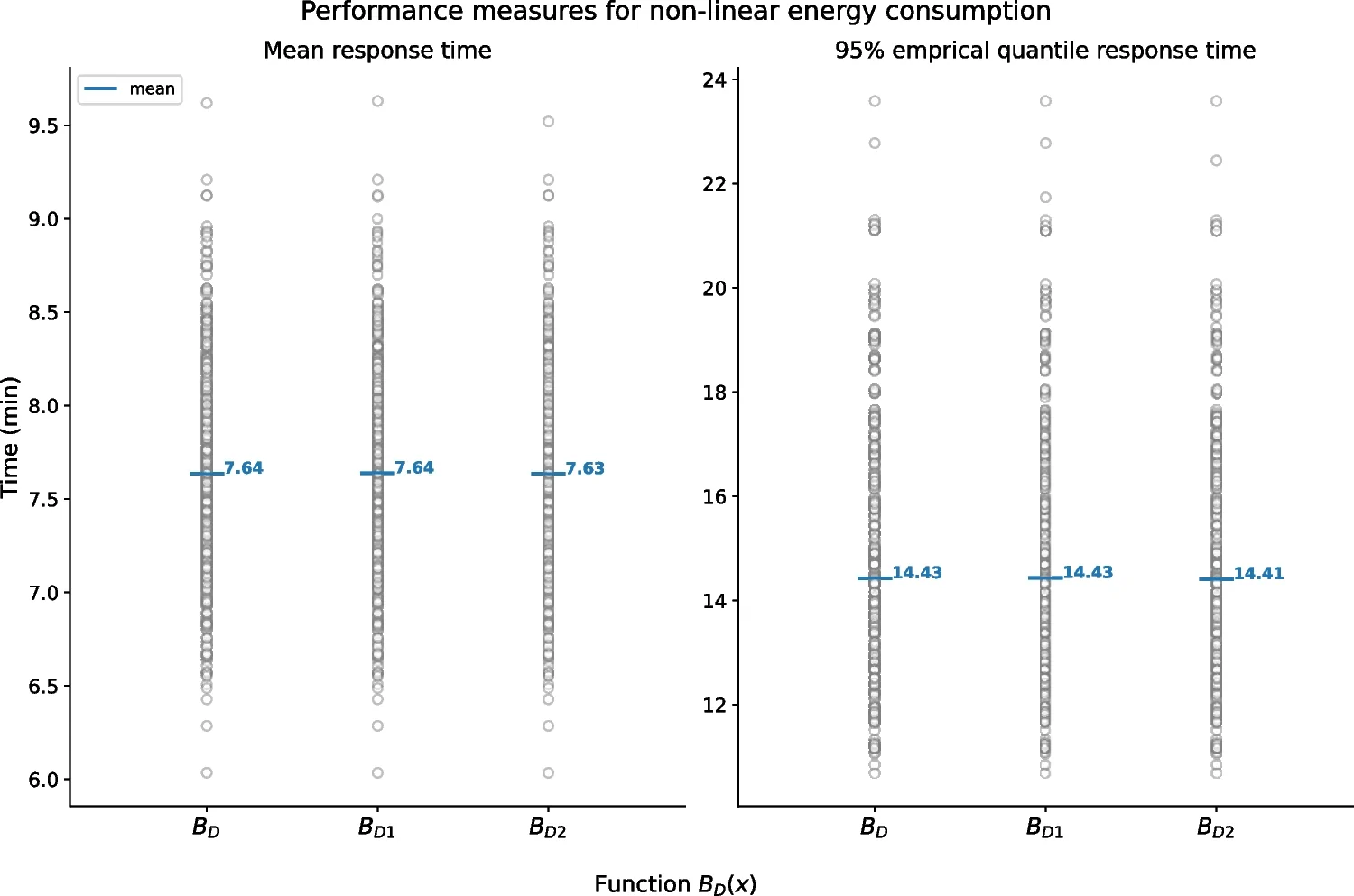
The mean and 95% empirical quantile of the response times for non-linear energy consumption and scenario RB1

We simulate both discharge functions with X=500 under charging scenario RB1. Using the parameters of the expected situation, the results are shown in Fig. 11. We observe that either function has a negligible impact under the expected operating conditions, indicating that the battery capacity and charging infrastructure are sufficiently generous that the precise shape of the energy-consumption function is of little importance.

**Fig. 12:**
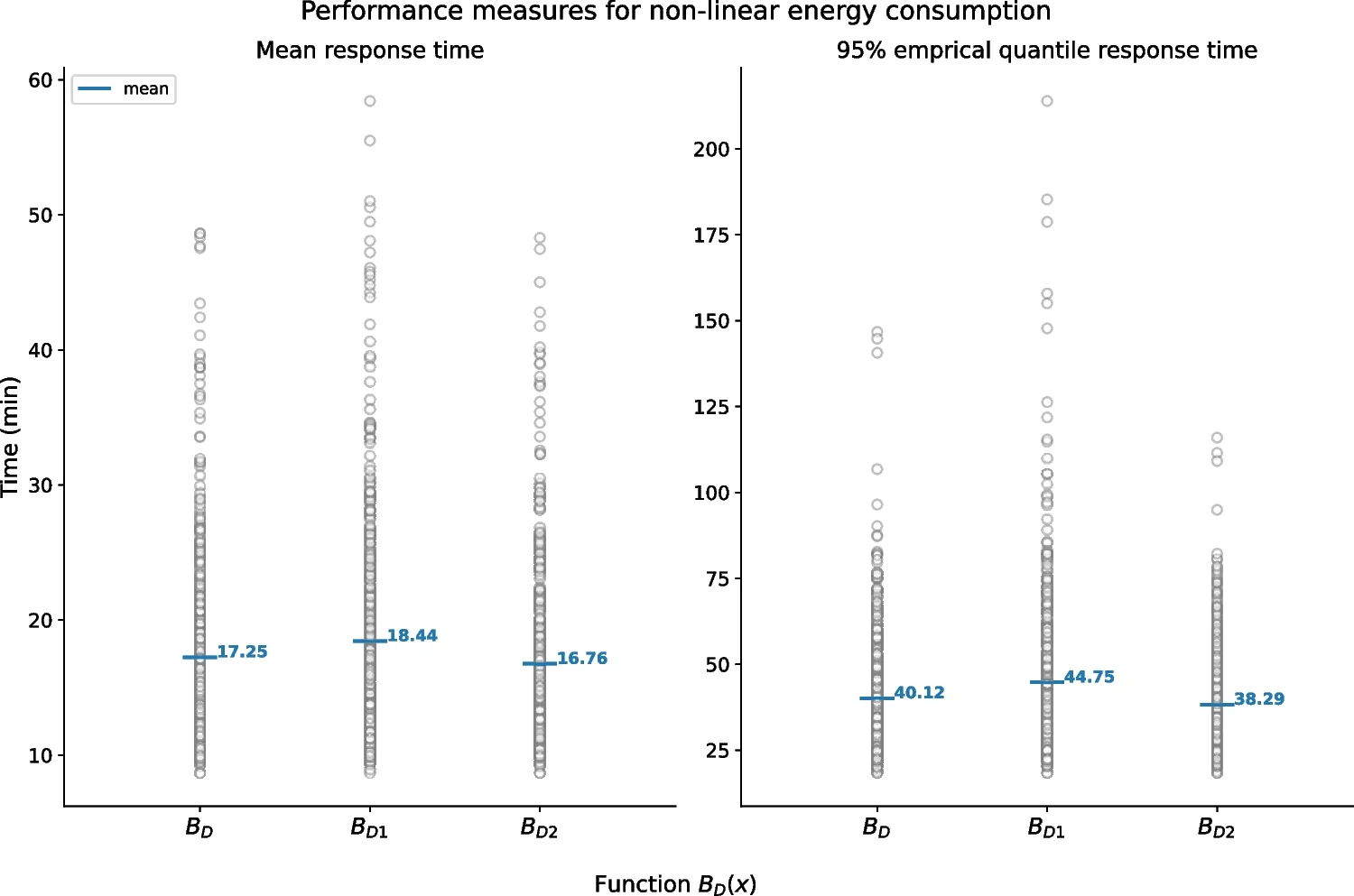
The mean and 95% empirical quantile of the response times for non-linear energy consumption, scenario RB1 and λ=1/4

We also set λ=1/4 to analyze what happens in busier scenarios: this is shown in Fig. 12. Here the choice of energy-consumption function becomes more influential. Function $B_{D1}$ results in somewhat longer response times, whereas $B_{D2}$ slightly improves performance relative to the constant model. This can be explained because in a busy scenario, ambulances are more frequently dispatched from more distant locations. $B_{D1}$ predicts higher energy consumption for longer distances than the constant model, causing ambulances to require charging more frequently, while $B_{D2}$ predicts the opposite. Nevertheless, the differences remain modest compared with the effects observed when battery capacity or charging infrastructure are varied.

### Simulation optimization

In Sections 4.1 and 4.2, we illustrated how ELASPY can be used to conduct scenario analyses and gain essential insights into the system’s performance. In this section, we show how ELASPY can be used to conduct a simulation-based optimization study.

In this optimization example, the goal is to find a distribution of chargers that achieves a comparable performance to a diesel fleet while minimizing the total number of chargers in the system. We consider the performance of two fleets equal when the confidence intervals of their 95%-QRT overlap.

**Fig. 13:**
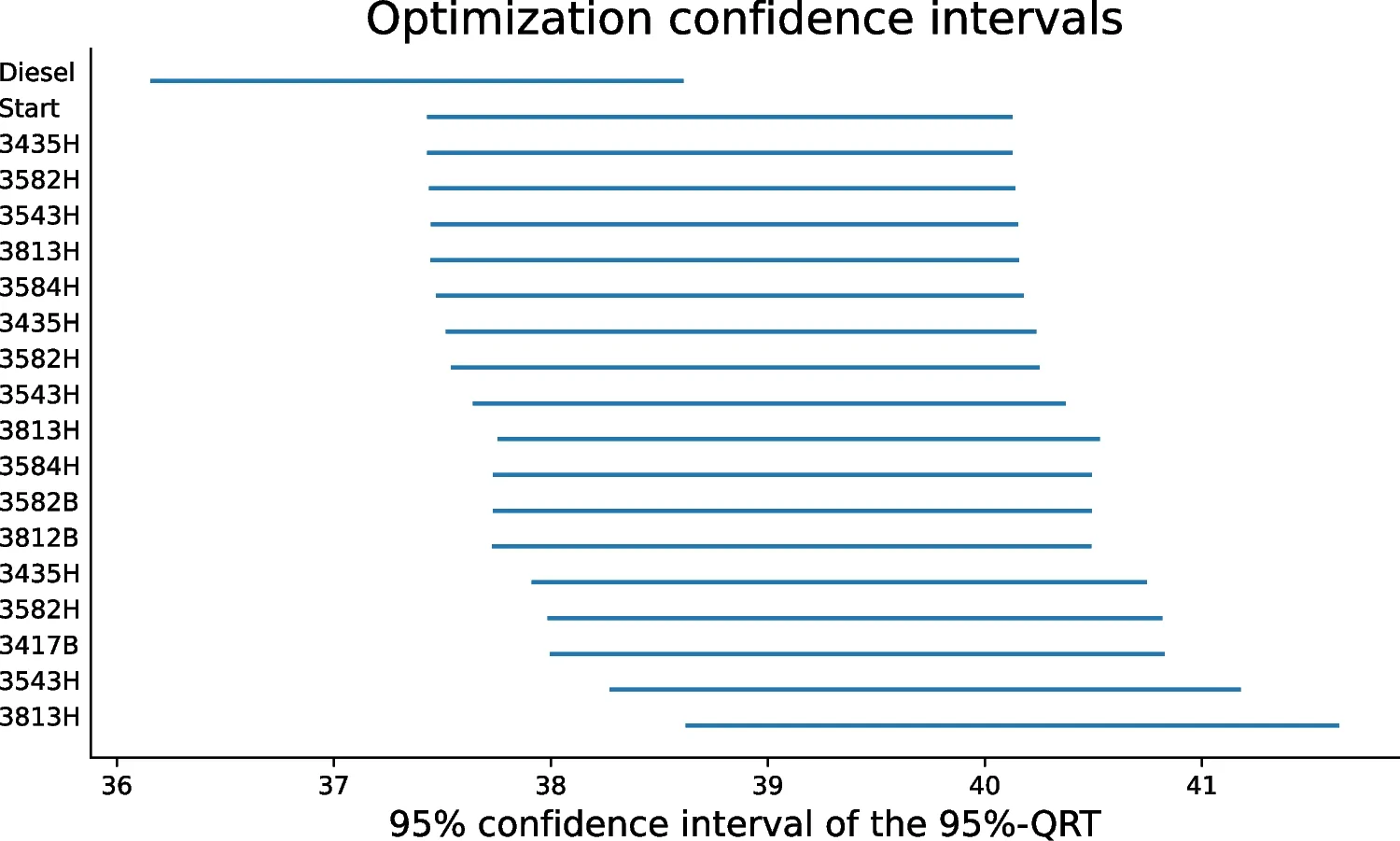
The development of the 95% confidence intervals of the 95%-QRT when executing the simulation-based optimization (Algorithm 2). Each line depicts one iteration - the removal of one charger. The charger at postal code 3543 was last to be removed: when attempting to remove more chargers (iteration 18, postal code 3813), the stopping criterion was met (the CI no longer overlapped with the CI for diesel)

To conduct this optimization, we use an iterative optimization scheme that is based on how many chargers are used in the system. As a measure for charger usage, we use the probability that at least one charger is available at a charging location. The higher this probability, the lower the charger usage at a location. In each iteration, we remove one charger at the location where this probability is the highest. The optimization method is summarized in Algorithm 2. Note that the minimum number of chargers at a base is equal to one.

**Algorithm 2:**
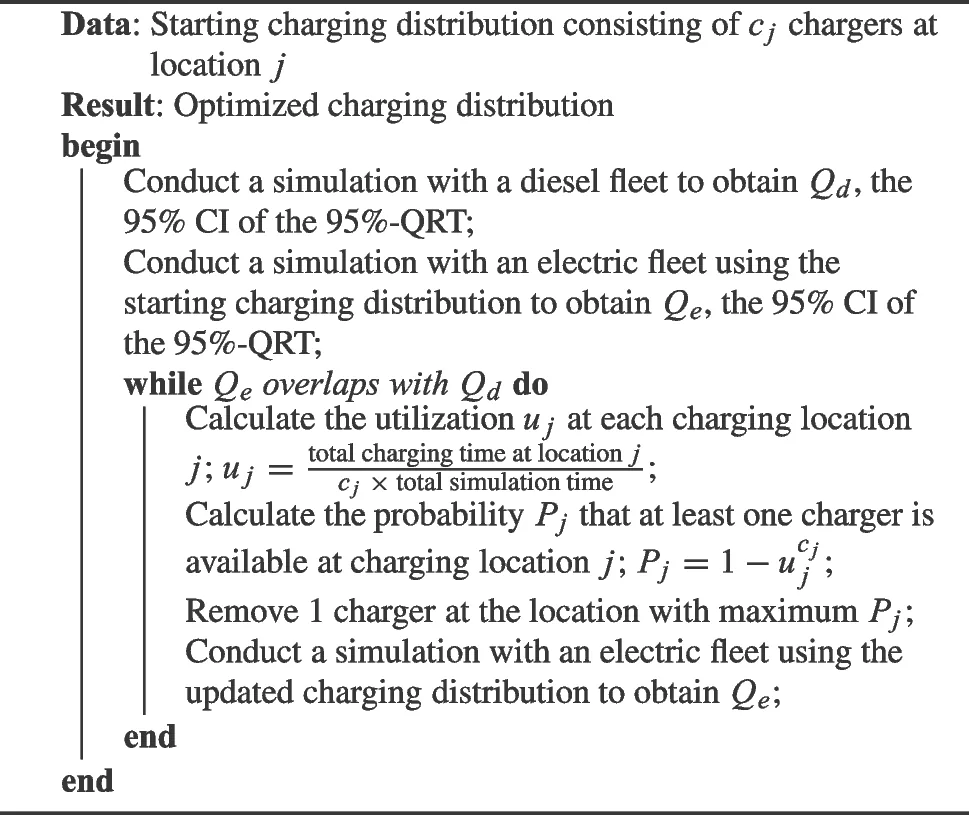
Pseudo-code for iteratively minimizing the number of chargers

To increase the busyness of the system sufficiently to obtain an interesting optimization case, we increase the arrival rate and reduce the battery capacity. We take λ=1/4 and B=125. We consider a charging scenario with only regular chargers, since fast chargers are usually more expensive and complex to place. Moreover, by only considering regular chargers, the system is further challenged due to lower charging speeds. In the starting charging station distribution, each hospital has 4 regular chargers and each base has a number of chargers equal to the number of stationed ambulances at that base. In total, this comes down to 40 chargers in the region. We run each simulation with X=500 simulation runs.

In total, 18 optimization iterations are required to optimize the number of charging stations in the system. Figure 13 shows the 95% confidence intervals ($Q_d$ and $Q_e$) for each iteration. The graph should be read from top to bottom. On the y-axis, the two uppermost lines represent the confidence intervals for the diesel and starting charging allocation. Next, the location is noted where one charger has been removed. In the first few iterations, chargers at the hospitals are removed with little to no influence on $Q_{e}$. Later in the optimization, the algorithm also removed chargers at bases. These particular bases were among those with the most charging stations. Interestingly, the influence of removing a single charger at a charging station is not the same in each iteration, since some removals have more or less influence. In total, 24 chargers turn out to be necessary to maintain overlapping 95% confidence intervals. In case of 24 chargers, three hospitals have one charger and two have two chargers. Moreover, bases that started with four chargers end up with three chargers, which indicates a pooling effect: at larger bases it is not always necessary to have the same number of chargers as ambulances.

To conclude, this section illustrates how ELASPY can be used in the context of simulation-optimization. Besides this example, ELASPY can also be used to solve other simulation-optimization problems, such as minimizing the total costs of a charging station allocation in a region.

### Managerial insights

In the previous sections, we have considered multiple numerical simulation experiments. By considering and analyzing all results, we derived some managerial insights. These will be discussed in this section.

First, the results suggest that the most limited charging scenario, RB1, is sufficient for maintaining a performance similar to that of a diesel fleet in normal circumstances, i.e., the expected situation. However, once circumstances become more demanding, the performance with the RB1 scenario deteriorates quickly with possibly life-threatening situations. Considering the high service levels ambulance providers should maintain, more charging stations are necessary to also be able to provide reliable aid in demanding situations. To determine where charging stations should be prioritized, the following insights based on our results for the province of Utrecht can be considered:

1. Having one fast charger at each base and one fast charger at each hospital (scenario FB1_FH1), achieves comparable performance to a diesel fleet, even in demanding circumstances.
2. Having one regular charger at each base and one fast charger at each hospital (scenario RB1_FH1) overall leads to lower response times than having one fast charger at each base and having one regular charger at each hospital (scenario FB1_RH1), indicating that fast chargers at hospitals add more value than fast chargers at bases.
3. If only chargers at bases can be placed, having one fast charger (FB1) results in lower response times than having two regular chargers (RB2).
4. With 150 kWh batteries, having one regular charger at every base and hospital (scenario RB1_RH1) is better than having one fast charger at each base (scenario FB1). However, with 115 kWh batteries, one fast charger at each base is preferred.
5. If fast chargers at bases are impossible, it is crucial in demanding situations that chargers are placed at the hospitals.
6. Hybrid fleets may provide an attractive intermediate solution during the transition to full electrification, mitigating many of the operational drawbacks of a fully electric fleet while still delivering part of the environmental benefits.

## Discussion and conclusion

Based on our case study, we expect that having one regular charger at each base is sufficient for daily operations in the province of Utrecht, the Netherlands. Our sensitivity analysis showed that when the system is approximately 50% busier (a busy fraction of 80%), response times do surge, much more so than with an equivalent diesel fleet. This may be relevant for periods of significantly increased demand, for example, during pandemic periods. The situation worsens if batteries with less capacity are purchased. In both cases, this might be alleviated to some extent by more and faster chargers.

The blow-up of the difference between diesel and electric response times for high busy fractions in Utrecht raises concern for other regions with high busyness. This should encourage them to use ELASPY as well. We recommend that they create a demand file using the times and locations of their historical incidents and feed this into ELASPY to evaluate the impact of transferring to electric ambulances. ELASPY works with generated data and with historical data, meaning that all demand patterns can be considered.

Although our results for the expected scenario are not a cause for concern, we suggest starting the transition with ambulances for non-urgent patient transport. This seems an uncontroversial approach [56]. In this context, patterns are much more predictable, and delays typically have less impact on patient outcomes. Such tests would provide valuable information regarding the variation in battery usage and the effectiveness of charging facilities. This information can help to calibrate the simulator and update the estimates before continuing the transition to urgent patient transport.

Ambulance providers may use different types of EMS operation rules than currently implemented in ELASPY. For example, a rule different from “assign the closest ambulance to a patient" may be used. To facilitate using different EMS operation rules, the code was written so that most functions perform a single action in the simulation, such as an ambulance driving to a patient or assigning an ambulance to a patient. This means that using a different EMS operation rule will usually require adapting a single function in the code. The elaborate documentation further facilitates implementing different rules. Moreover, the framework can also be extended so that the number of vehicles can vary throughout the day. ELASPY works with a list of ambulances that can be adjusted dynamically by adding or removing ambulances during the simulation. If an ambulance needs to be removed, one should add some simple logic about what happens when the ambulance is busy. For example, a logical approach would be to let the ambulance finish helping the current patient and then let it drive back to its base upon which it is removed from the ambulance list. If patients arrive in the meantime, one should ignore this ambulance for dispatch.

### Limitations and discussion

We next discuss the assumptions underlying our case study. First, we use centroids of postal codes as proxies of locations. However, distances used in the simulator are somewhat realistic as they are not based on Euclidean distances, but on the road network. Second, we do not consider day and night patterns in arrivals. This choice is strengthened by the observation that ambulance demand is larger during the day than during the night and, therefore, the most pressing issues are expected towards the end of the day. This was also considered to be realistic by Dutch industry experts. For this reason, our experiments considered the performance during daytime. Third, we did not consider interregional rides. These are rides between regions that occur infrequently: less than 2 per day in Utrecht Ambulancezorg Nederland [4, p.71]. Fourth, our scope excluded non-urgent, plannable patient transport. We did not see a generic approach to this, as whether or not these transports are executed by the same vehicles that serve urgent patients, differs per ambulance provider. Fifth, we did not model lunch breaks, crew changes or unplanned events such as vehicle malfunction. Finally, recharge and discharge rates were modeled as deterministic. In principle, ELASPY can readily accommodate stochastic discharge rates, provided the assumed distribution has a finite upper tail. This would primarily affect dispatch decisions, since the simulation only dispatches an ambulance if it is expected to have sufficient remaining range to complete the trip without becoming stranded. Under a stochastic discharge model, this deterministic range check should instead be replaced by a criterion based on an appropriate upper quantile of the energy-consumption distribution (or another suitably conservative bound).

### Further research

Although this paper primarily addresses strategic planning, the electrification of an ambulance fleet also introduces important operational challenges. For instance, how should the SoC be integrated into dispatch decision-making? Consider a scenario in which two vehicles at the same base are both sufficiently charged to respond to an emergency. Should the vehicle with the higher SoC be dispatched, or might it be preferable to deploy the one with the lower SoC, thereby retaining a vehicle with greater remaining range for future calls? Additionally, the integration of SoC and real-time charger availability into dynamic relocation strategies presents further complexity. These operational considerations represent substantial research questions in their own right and warrant dedicated investigation.

We next list some avenues for future research that we deemed irrelevant for the Netherlands, but that might be useful for regions that operate under different circumstances. For example, ambulance providers that face targets that are very hard to meet or with a very high busy fraction, leaving little time for charging, might require many expensive fast chargers. In such a case, it may be useful to perform a simulation-based optimization study that allocates chargers to locations in order to minimize, for example, total cost. ELASPY can also be an aid in such a study, as illustrated in Section 4.3.

Another scenario might be that an ambulance provider is so busy overnight that our assumption of starting each day with a fully charged fleet becomes unrealistic. This could be handled by extending ELASPY: at the beginning of each day, sample the SoC of the fleet from a distribution that matches the observed SoC distribution of the fleet at the end of the day. Besides these ideas that we did not think were worth pursuing for the Netherlands, we end with a few recommendations that we consider relevant for any region.

We encourage finetuning the estimates for ambulance energy consumption. ELASPY currently models energy consumption deterministically based on the expected value according to industry experts. We deemed this realistic enough, based on Galvin [23, Figure 4]. However, if future users were to have data on how ambulance speed varies during trips, implementing a more detailed relation between energy consumption and speed would be a natural extension of ELASPY. Another, more sophisticated way of estimating energy consumption is via automotive data loggers that can be attached to operational diesel ambulances.

Another avenue for future research is investigating the influence of smart charging strategies. These strategies can, for example, balance the grid capacity which prevents peaks in power usage. This does mean that the charging capacity varies over time, which would impact how much an ambulance can charge. Smart strategies can also allocate different power levels to different ambulances, so that, for example, ambulances with a lower SoC can be charged with more power. Finally, the transition requires a careful consideration of large calamities, such as prolonged power outages. Ambulancezorg Nederland [5] suggests searching for a balance between owning the chargers for daily use and adding a flexible shell of third-party chargers, that give priority to EMS in extreme situations.

## Figures and Tables

**Fig. 14:**
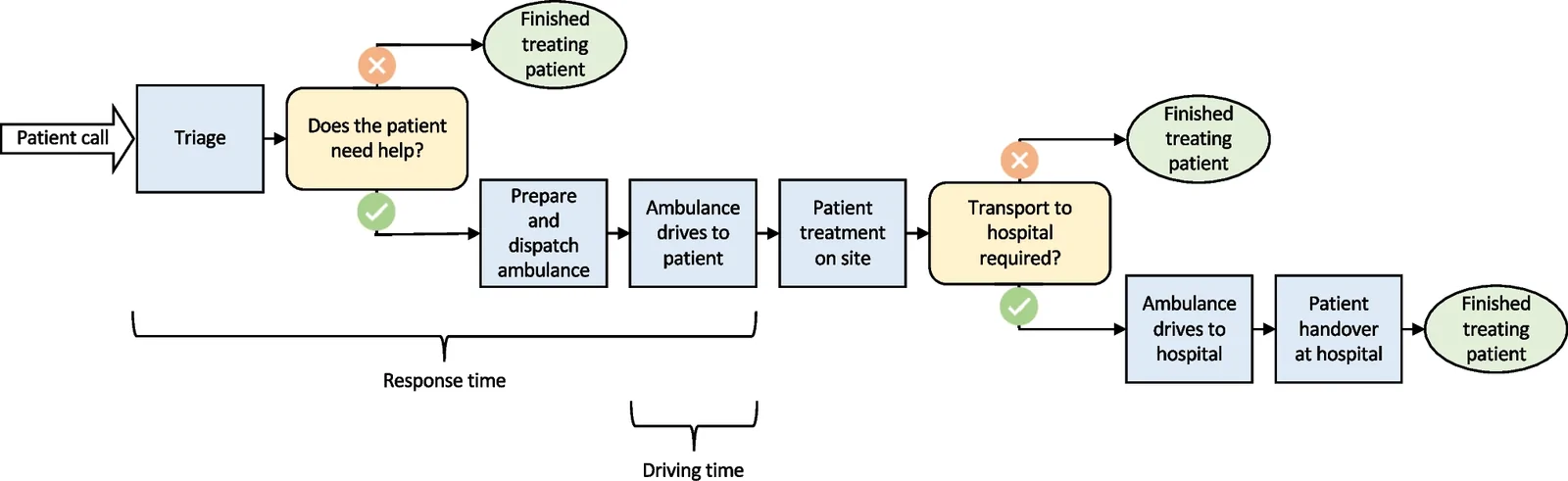
Overview of the emergency response process

**Fig. 15:**
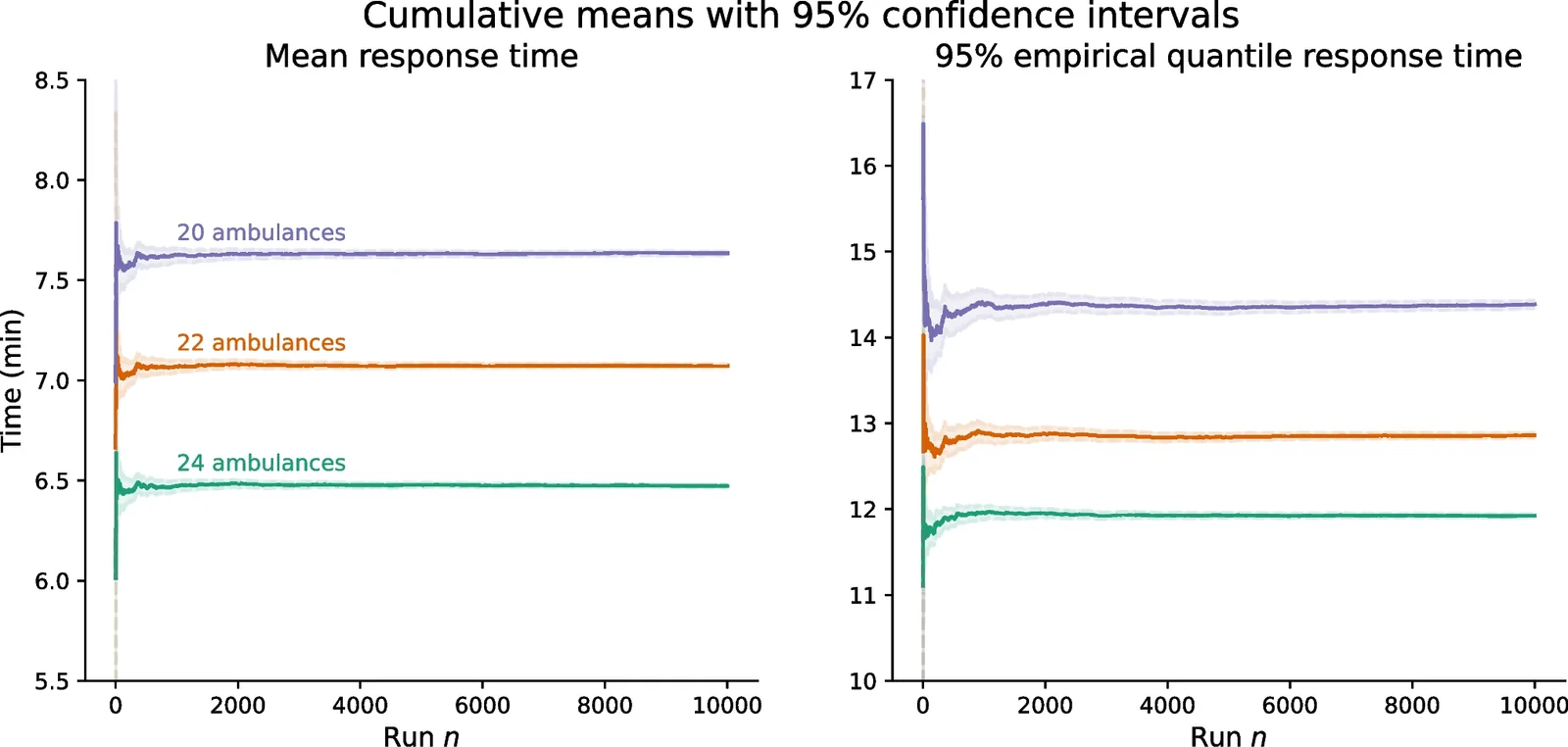
The cumulative means of the two performance measures (mean and 95% empirical quantile) with 95% confidence intervals

**Fig. 16:**
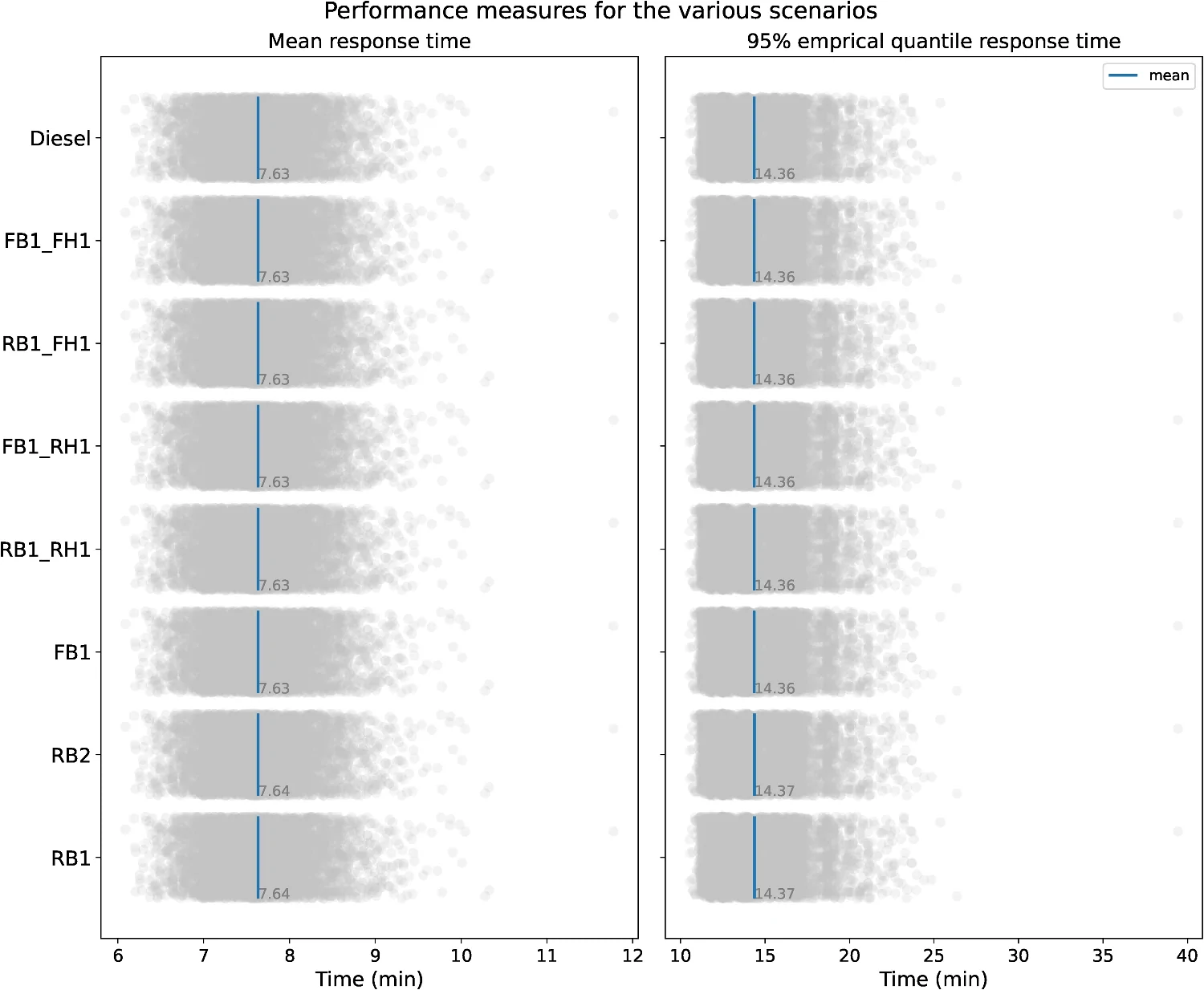
The mean and 95% empirical quantile of the response times of all scenarios for X=5000 simulation runs

**Fig. 17:**
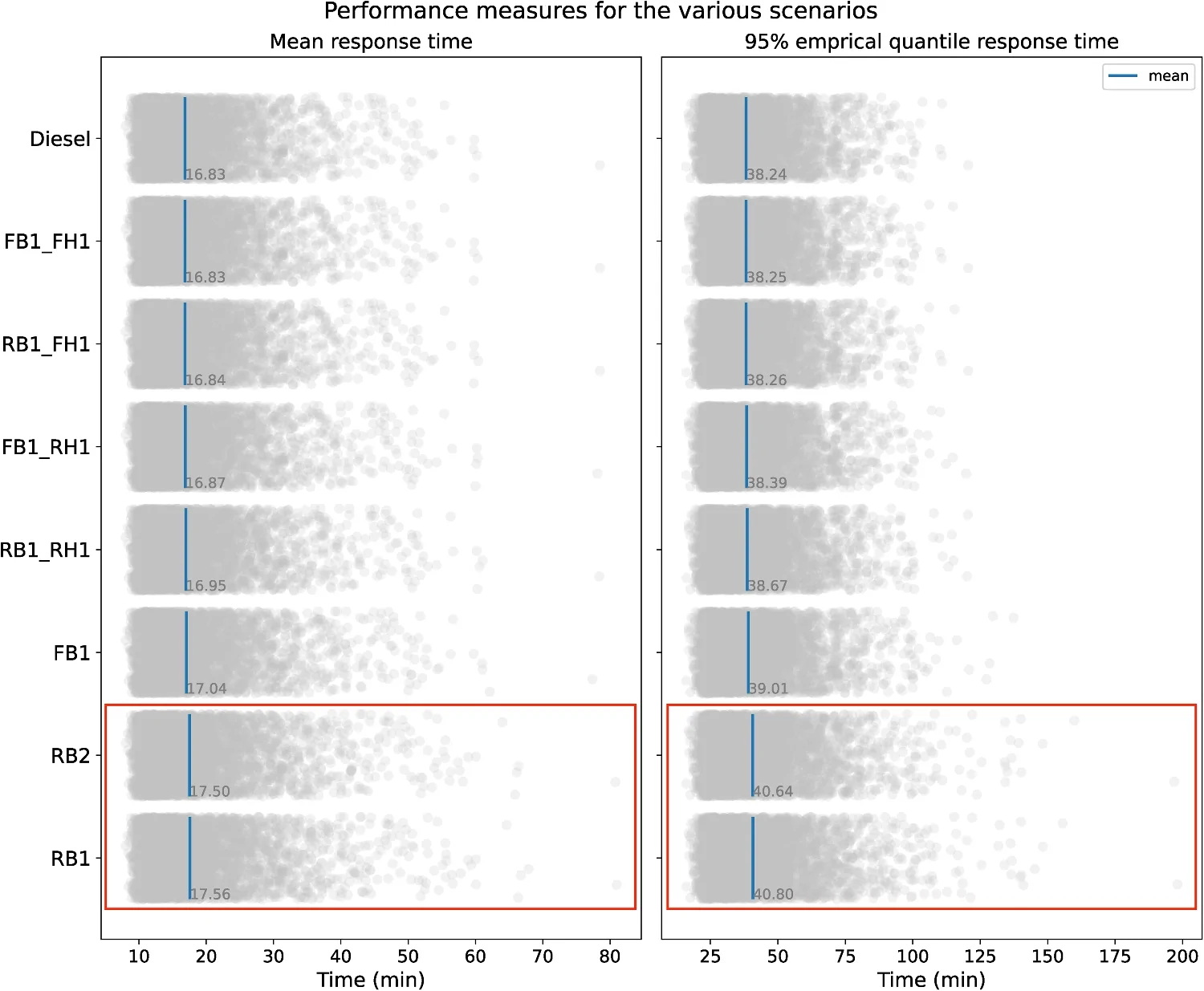
The mean and 95% empirical quantile of the response times of all scenarios for λ=1/4 and X=5000. The red rectangles indicate the scenarios where the 95% confidence intervals of the performance measures do not overlap with those of the diesel case

**Fig. 18:**
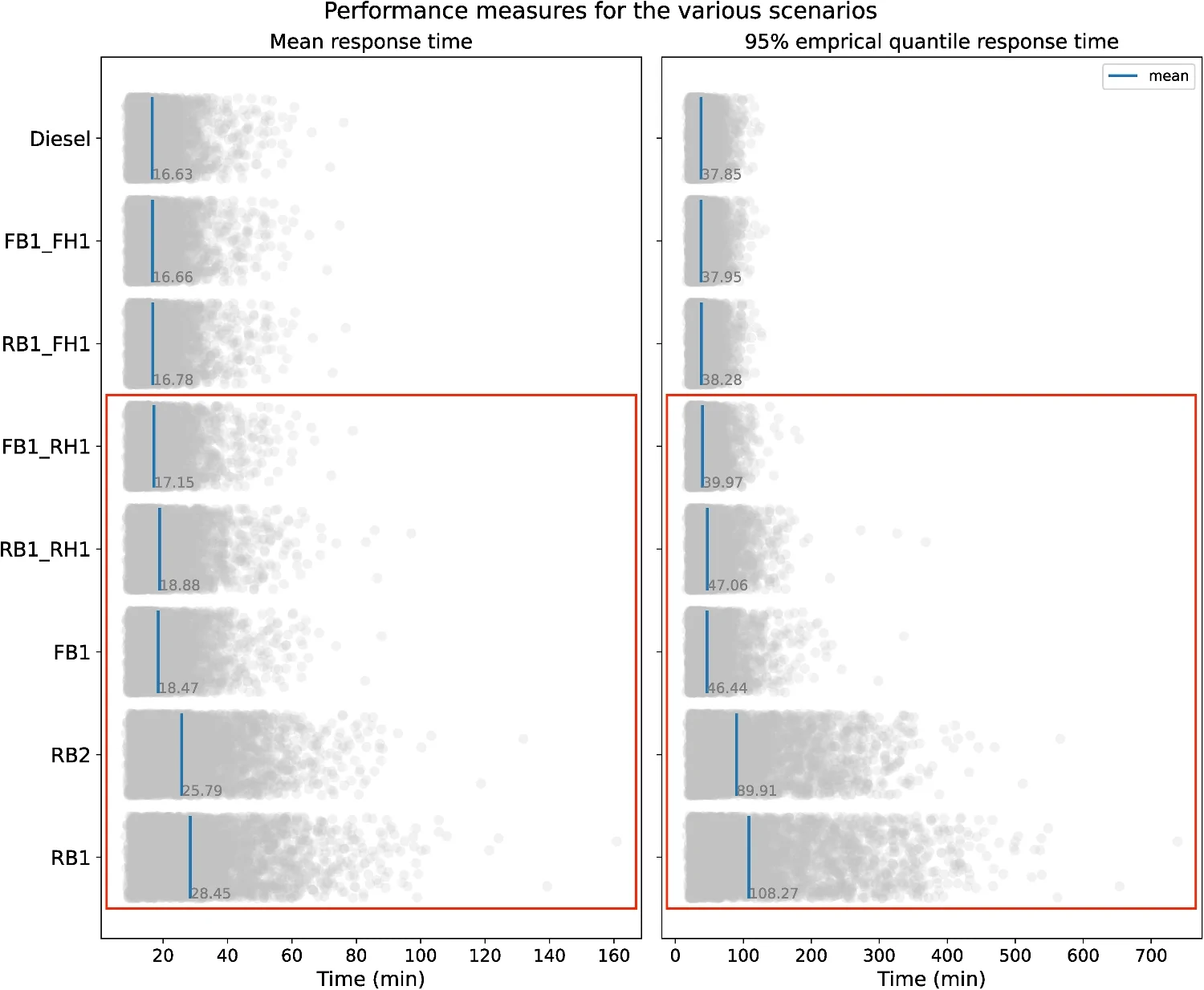
The mean and 95% empirical quantile of the response times of all scenarios for λ=1/4, B=115 and X=5000. The red rectangles indicate the scenarios where the 95% confidence intervals of the performance measures do not overlap with those of the diesel case

## Data Availability

Our manuscript has associated data in a data repository: https://github.com/NanneD/ELASPY

## A Appendix methods and data

### A.1 Emergency response process

This paper focuses on urgent ambulance demand that is not plannable in nature. Figure 14 presents an overview of the corresponding emergency response process. At the moment an emergency call arrives in the call center, three minutes are reserved for handling the call and the ambulance crew getting ready [4, p.78] (‘tijdsduur verwerking MKA’ +‘ tijdsduur uitrukken’). The dispatcher decides which ambulance drives to the patient. If none are available, the patient has to wait until one becomes available. Once the ambulance arrives at the incident scene, the patient is diagnosed and potentially treated on-site. The crew decides whether the patient also requires transportation to a hospital, and if so, executes the additional ride. Upon arrival there, the crew transfers the patient to the hospital staff. After this handover, the ambulance becomes available again for new patients. If no patients are waiting, the ambulance returns to its base. In the case of electric ambulances, the process is the same, although the ambulance has the option to charge at certain locations. This is further addressed in Section 3.

### A.2 Number of simulation runs

As explained in Section 3.5, the number of performed simulation runs (*X*) influences the running time, but also the accuracy of the performance measure estimates. In order to balance running time and accuracy, we conducted an experiment to determine the value of *X*.

We performed 10,000 simulation runs for 20, 22 and 24 diesel ambulances. The ambulances are distributed over the bases according to MEXCLP (see Section 3.5). With each run, we updated the mean estimate of the two performance measures (also known as the cumulative mean) and their confidence intervals. Figure 15 presents these results. The estimates stabilize after a few thousand runs and a single run takes approximately 0.21 seconds on a 2.3 GHz 8-Core Intel Core i9 with 16GB of RAM laptop. Based on these results, we take X=5000 for most experiments; if we use a different value, this is specified explicitly.

## Footnotes

1 In RAVU, 95% of the A1 calls are served in 17:21 minutes [4, p.78] but this includes 3 minutes of call handling, so we use 14:21 minutes).

2 The 95%-confidence interval of the 95%-QRT of response times.
